# Revision of genus *Pericalus* from China, with descriptions of four new species (Carabidae, Lebiini, Pericalina)

**DOI:** 10.3897/zookeys.758.24169

**Published:** 2018-05-14

**Authors:** Hongliang Shi, Hongbin Liang

**Affiliations:** 1 College of Forestry, Beijing Forestry University, Beijing 100081, China; 2 Key laboratory of Zoological Systematics and Evolution, Institute of Zoology, Chinese Academy of Sciences, Beijing 100101, China

**Keywords:** Carabidae, China, *Pericalus*, new species, key

## Abstract

Nine taxa of the genus *Pericalus* Macleay from China are revised, with four new species described: *Pericalus gibbosus* **sp. n.** (type locality: Putao, Myanmar; one paratype from Mêdog, Xizang), *Pericalus elegans* **sp. n.** (type locality: Mêdog, Xizang), *Pericalus acutidens* **sp. n.** (type locality: Longchuan, Yunnan), and *Pericalus obscuratus* **sp. n.** (type locality: Fanjingshan, Guizhou). Four taxa are newly recorded from China: *Pericalus ornatus ornatus* Schmidt-Göbel (Yunnan, Hainan), *P. obtusipennis* Fedorenko (Yunnan), *P. amplus* Andrewes (Yunnan), and *P. dux* Andrewes (Yunnan). *Pericalus formosanus* Dupuis is newly ranked as a subspecies of *P. ornatus* Schmidt-Göbel. An improved key to world species of the subgenus Pericalus is provided, along with distribution maps and images of habitus and male and female genitalia for all Chinese species.

## Introduction

The genus *Pericalus* belongs to the subtribe Pericalina (Carabidae: Lebiini), distributed in the tropical regions of Oriental-Australasian Realm: the easternmost reaches New Ireland (*P. novaeirlandiae*), and the westernmost reaches south India (*P. fascinator*). *Pericalus* is close to the following four Oriental genera: *Lioptera* Chaudoir, *Coptodera* Dejean, *Trichocoptodera* Louwerens and *Gidda* Andrewes. These five genera belong to the *Pericalus* genus-group (Shpeley and Ball 2000) in having the following three character states: mentum without a median tooth, palpifer with one long seta, and the terminal labial palpomere fusiform.

Two distinct subgenera in *Pericalus* are recognized, distinguished by their different pronotal characters. The subgenus Coeloprosopus, including 20 described species, was well documented with a recent complete review (Baehr 1994), and two additional species described later (Baehr 2000a, 2003). In contrast, study on the subgenus Pericalus was relatively weak, only with incomplete keys published (Dupuis 1913, Andrewes 1937, Jedlička 1963, Baehr 2000b) until 2017. When preparing an early draft of the present paper, a study focusing on Vietnamese *Pericalus* species with a key for all known species in the subgenus was published (Fedorenko 2017). However, species determination in this subgenus is still not clearly solved; for example, some species lack clear definition and diagnoses, and *P. formosanus* *sensu* Fedorenko, 2017 actually belongs to a new species also distributed in China.

The previous study of *Pericalus* from China was very inadequate. Only one species, *P. formosanus*, was recorded in Taiwan, and the fauna in the Chinese continent is almost unreported (Kabak 2003). Our examined material shows that the *Pericalus* beetles are common and diverse in tropical regions of China (south Yunnan, Hainan, south Xizang). During recent expeditions to Guizhou, Guangxi, south Xizang, and north Myanmar, three very rare and narrowly distributed new species were collected. After detailed morphological study, all specimens from China were identified, with four new records for the county. Moreover, one common species from southwest Yunnan, south Xizang, and north Myanmar previously determined as *P. ornatus* is attributed to a new species, *P. acutidens* sp. n. *Pericalus ornatus formosanus* is newly ranked as a subspecies based on the almost identical male genitalia and slightly external differences with the nominal typical subspecies. Thus the fauna of *Pericalus* in China has eight species and one subspecies in total, all belonging to the subgenus Pericalus.

The present paper aims to review all nine taxa of the genus *Pericalus* from China, with the descriptions of the four new species, and to provide an improved key to accommodate all known taxa of *Pericalus* (*s. str.*) but two dubious and little known species, which are discussed.

## Materials and methods

This work is based on the examination of 218 specimens from China and some other Southeast Asian counties. A total of 19 taxa of the subgenus Pericalus were treated, with 15 of them examined. Examined material for species outside of Chinese fauna were cited as well. Most specimens are from the collection of IZAS. Collections cited in the present paper are indicated by the following abbreviations:

**CCCC** Collection of Changchin Chen, Tianjin, China

**CRS** Collection of Riccardo Sciaky, Milano, Italy

**IRSN** Institut Royal des Sciences Naturelles, Brussels, Belgium

**IZAS** Institute of Zoology, Chinese Academy of Science, Beijing, China

**NHML** The Natural History Museum, London, United Kingdom

**NMPC** Národní Muzeum Přírodovědecké Muzeum, Prague, Czech Republic

**NNML** Naturalis Nationaal Natuurhistorisch Museum, Leiden, Netherland

**OUM** Oxford University Museum of Natural History, Oxford, UK

**SNU** Shanghai Normal University, Shanghai, China

**ZMMU** Zoological Museum of the Moscow State University, Moscow, Russia

**ZSM** Zoologische Staatssammlungen, München, Germany

Body length was measured from the apical margin of the labrum to the elytral apex; head width (**HW**) was the greatest width including eyes; pronotum length (**PL**) was measured along its median line; and pronotum width (**PW**) was the greatest width of the pronotum; elytra length (**EL**) was measured from elytral base to apex; elytra width (**EW**) was the combined width of each elytron at its widest points; **D2** was the distance of the second elytral discal setigerous pore to elytra base. All measurements were based on examined materials only. Details of methods for dissection, and terminology follow our previous work (Shi et al. 2013). Distribution maps were created in Adobe Photoshop software based on examined materials and confirmed records from literature. Unconfirmed records from literature are marked by empty circles in the maps.

## Taxonomy

### Subgenus Pericalus (s. str.)

Taxon classificationAnimaliaColeopteraCarabidae

Macleay, 1825

Macleay 1825: 15. Type species: Pericalus cicindeloides Macleay, 1825, by monotypy.

#### Diagnosis.

The genus *Pericalus* is distinguished from other genera of Pericalina by the combination of the following characters: dorsal surface glabrous, except for the eighth and ninth elytral intervals which are generally sparsely and very finely setose; black or metallic in color, elytra either unicoloros or with two groups of yellowish patches; eyes strongly prominent; labrum elongate, apex deeply notched; clypeus with apical margin straight; terminal labial palpomere fusiform in both sexes; labial palpifer with one long seta; mentum without median tooth; paraglossa membranous, longer than ligula; third interval of elytra with two to four setigerous pores, the first one near base, the last one very close to apex; fourth tarsomere simple; claws smooth.

The subgenus Pericalus is different from the subgenus Coeloprosopus Chaudoir by its body size being usually larger; in having the lateral channel of the pronotum widely explanate, with the lateral bead indistinct; and the pronotum subequal to the width of head across eyes.

#### Distribution.

The subgenus Pericalus containing 19 taxa has an Asia mainland-Sundaland distribution pattern. More than half of them (eleven taxa) are distributed through the Asian continent tropical-subtropical areas, and the most diverse region is located in northern Myanmar, northeast India, southeast Xizang, and west Yunnan. The remaining eight taxa are distributed in the Sundaland region (Greater Sunda Islands and Malay Peninsula). There are no species endemic to the Philippines or islands in the Wallacea region, but one species is widely distributed in each region, *P. cicindeloides* in the Philippines, and *P. baehri* in Sulawesi.

#### Habitat.

Many adults of the subgenus Pericalus were collected in daytime under barks of fallen logs in tropical or subtropical forests. Some others were collected during night, fast running on surfaces of dead logs, or occasionally attracted by lights. Species in this subgenus usually have a rather flat habitus, adapting for living under tree bark.

#### Taxonomic characters.

The most important taxonomic characters in this subgenus are: (1) dorsal coloration, including elytral pattern; (2) number of setigerous pores on the third interval; (3) shape of elytral outer apical angles; and (4) shape of elytral sutural apical angles.

The supposed ground plan for the subgenus is supposed to include two groups of yellowish patches on the elytron (unicolorous in four species); body very flat (but convex in *P. gibbosus*); elytra with microsculpture consisting of distinct, very transverse meshes (but nearly isodiametric in *P. dux* and *P. elegans*); outer apical angles of elytra distinct, acuminate to rounded; elytral sutural angles sharp or blunt; third interval of the elytra with three setigerous pores (but four pores in four species, usually two in *P. o. formosanus*).

The shape of outer apical angles of elytra can be classified into the following four basal forms: **acuminate**, apex forming a very sharp angle, strongly projecting, less than 90 degrees (Fig. 21), present in seven species; **acute**, apex forming a sharp and distinct angle, not or weakly projecting, more than 90 degrees (Figs 22, 24), in two species; **obtuse**, apex forming an indistinct angle, more than 90 degrees (Figs 23, 25), in five species; **rounded**, apex fully rounded, not angulate (Figs 26–32), in seven species. There are some taxa, such as *P. o. formosanus*, with one form of outer apical angle in general (obtuse), but very rarely other forms can be present in a few individuals (rounded or acute).

Most taxa (15 of 19) of the subgenus have two, the anterior and posterior, groups of yellowish patches on elytra; variation includes four and two pattern forms respectively. For different species or individuals belonging to same pattern form, pale markings on certain intervals may be obsolete or their positions moved.

**Figures 1–6.**
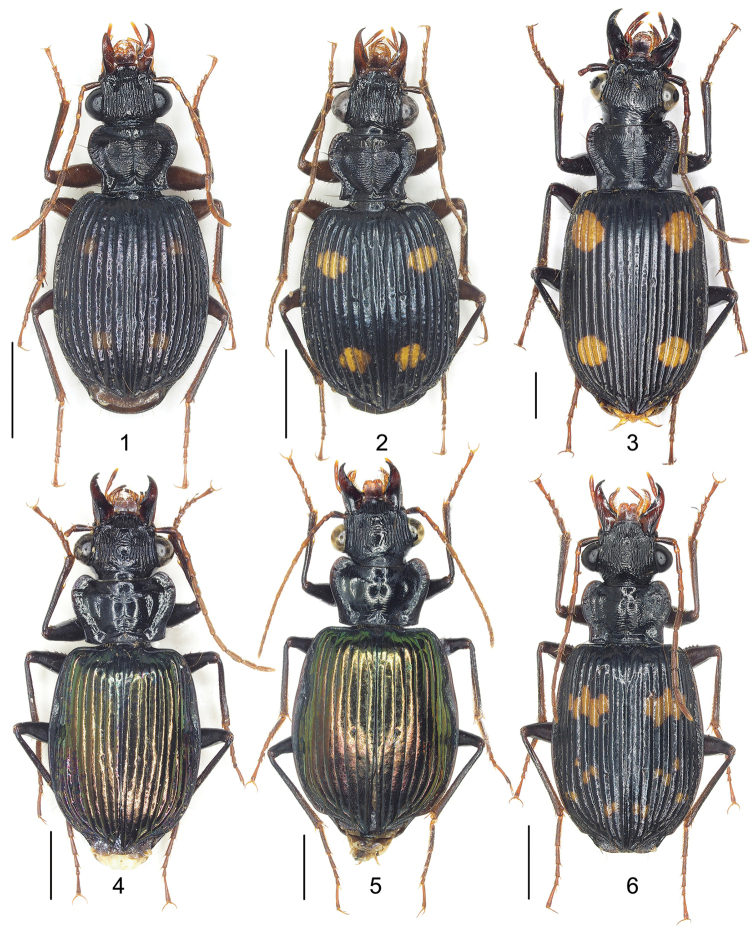
Habitus **1** *P. gibbosus* sp. n., holotype, Myanmar (Putao), male **2** *P. gibbosus* sp. n., paratype, Myanmar (Putao), female **3** *P. dux* Andrewes, Yunnan (Jinghong), female **4** *P. elegans* sp. n., holotype, Xizang (Mêdog), male **5** *P. elegans* sp. n. paratype, Xizang (Zayü), female **6** *P. amplus* Andrewes, Myanmar (Putao), male. Scale bars: 2 mm.

**Figures 7–12.**
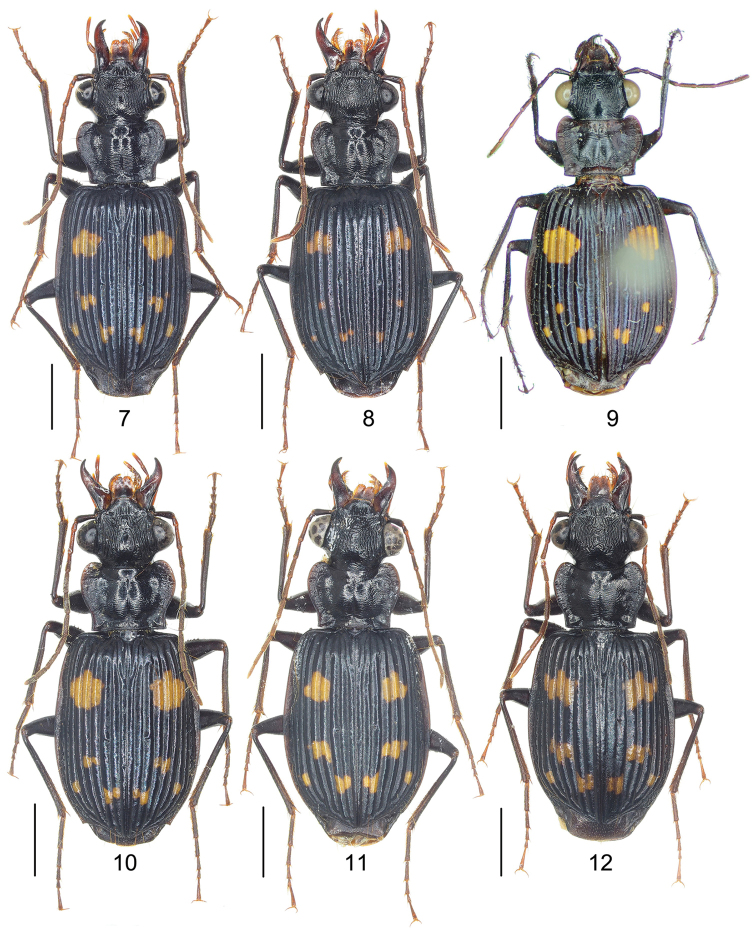
Habitus **7** *P. acutidens* sp. n., holotype, Yunnan (Longchuan), male **8** *P. acutidens* sp. n., paratype, Myanmar (Putao), female **9** *P. ornatus ornatus* Schmidt-Göbel, syntype, Myanmar (Tenasserim), male **10** *P. ornatus ornatus* Schmidt-Göbel, Yunnan (Hekou), male **11** *P. ornatus ornatus* Schmidt-Göbel, Hainan (Baisha), male; **12** *P. ornatus formosanus* Dupuis. Taiwan (Yilan), female. Scale bars: 2 mm.

**Figures 13–15.**
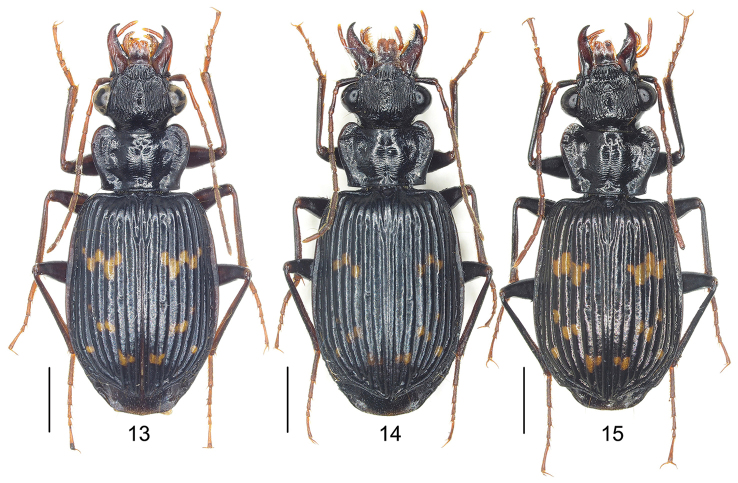
Habitus **13** *P. obscuratus* sp. n., holotype, Guizhou (Fanjingshan), male **14** *P. obscuratus* sp. n., paratype, Guizhou (Fanjingshan), female **15** *P. obtusipennis* Fedorenko, Yunnan (Pianma), male. Scale bars: 2 mm.

There are four basic forms for anterior patches: **round**, in six taxa. Usually a single large round or nearly round spot (Figs 1–3, 7–11) occupies some of the third to eighth intervals, two to five intervals in width. In some individuals of *P. ornatus* (Fig. 12), the spot is somewhat transverse and irregular, similar to the zigzag form, but generally less transverse and with less displacement of odd and even pale markings. **Zigzag**, in five species. A transverse serrated band occupies the third to seventh intervals (Figs 6, 13–15), with pale markings on odd intervals anteriorly placed and those of even intervals posteriorly placed. Sometimes pale markings on the third and/or seventh intervals are obsolete. In *P. baehri* pale markings on the third interval are placed much more anteriorly to those on the remainder of the odd intervals. **Separated**, in two species. The basic pattern includs five small separated spots, only one interval in width for each, placed on the third to seventh intervals respectively. Sometimes spots on the sixth and seventh intervals are obsolete. Longitudinal positions for all spots are similar to those of the zigzag pattern. **Double**, a special pattern only for *P. cordicollis*. The double pattern form is composed of two adjacent large spots: the inner one is in the third and fourth intervals, posteriorly placed; the outer one is in the fifth to eighth intervals, anteriorly placed.

Two basic forms exist for posterior patches: **separated**, in eleven taxa. The basic pattern is composed of three separate spots (Figs 7–12): the first one is in the second and third intervals, posteriorly placed; the pale marking on the second interval is occasionally obsolete. The second one is in the fourth to sixth intervals, anteriorly placed; the pale marking on the fourth interval is occasionally obsolete; the pale marking on the sixth interval is adjacent to that on the fifth (*P. cordicollis*, and some *P. ornatus* and *P. acutidens*, Figs 7, 11, 12), placed much anteriorly to that on the fifth and forming a small separate spot (*P. amplus*, *P. obtusipennis*, *P. obscuratus*, Figs 6, 13–15), or obsolete (the remaining four species and most *P. ornatus* and *P. acutidens*, Figs 8–10). The third one is in the seventh and eighth intervals, posteriorly placed, sometimes a little before the first spot; the pale marking on the eighth interval is usually obsolete, but present in *P. cordicollis*, and some individuals of some other species. **Single**, in three species. The basic pattern is a single large round spot in some of the second to seventh intervals, two to four intervals in width (Figs 1–3). In a few individuals of *P. longicollis*, the pale marking on the third interval is present and placed posteriorly to the main spot, but is obsolete in most individuals.

#### Key to the world species of the subgenus Pericalus

**Table:** 

1	Third elytra interval with four setigerous pores	**2**
–	Third elytra interval with two or three setigerous pores	**4**
2	Elytra dark with two groups of yellowish patches, the anterior one zigzag, the posterior one separate; elytral outer apical angles rounded; India, Myanmar, Vietnam, China (Yunnan)	***P. amplus* Andrewes**
–	Elytra unicolorous, black or metallic, without yellowish patch; elytral outer apical angles acute, or obtuse	**3**
3	Dorsal surface with strong metallic luster, bluish, greenish or violaceus; Borneo, Sumatra, Java, Malay Peninsula, the Philippines	***P. cicindeloides* Macleay**
–	Dorsal surface black, with very faint purplish luster; India	***P. fascinator* Andrewes**
4	Elytral sutural angles spiniform; the middle setigerous pore placed posterior to the midpoint of elytra (D2/EL > 0.52)	**5**
–	Elytral sutural angles blunt; the middle setigerous pore placed anterior to the midpoint of elytra (D2/EL < 0.46) or rarely lacking	**9**
5	Elytra cupreous green, without patch; elytral outer apical angles rounded (Fig. 31); China (Mêdog, Zayü)	***P. elegans* sp. n.**
–	Elytra dark with yellowish patch; elytral outer apical angles acuminate (as in Fig. 21); Malaysia, Indonesia	**6**
6	Elytral anterior patch round or zigzag, forming single continues patch	**7**
–	Elytral anterior patch separate, divided into three to five small spots	**8**
7	Elytra metallic blue or green; posterior patch single; Sumatra, Borneo, Malay Peninsula	***P. longicollis* Chaudoir**
–	Elytra metallic blue-violaceus; posterior patch separate, composed of three to five small spots; Sumatra, Java, Borneo, Sulawesi	***P. baehri* Fedorenko**
8	Elytra metallic blue-violaceus; Malay peninsula	***P. violaceus* Andrewes**
–	Elytra black, at most with faint metallic luster; Sumatra, Java, Bali	***P. guttatus* Chevrolat**
9	Elytral outer apical angles acuminate, acute or obtuse (Figs 21–25)	**10**
–	Elytral outer apical angles rounded (Figs 26–32)	**14**
10	Elytral anterior patch composed of two large spots, sometimes partly conjoint; Borneo	***P. cordicollis*** Andrewes
–	Elytral anterior patch round or zigzag form, forming single continues patch	**11**
11	Elytral lateral border strongly incised near anterior third and then expanded; anterior patch strongly transverse and zigzag; Sumatra	***P. funestus* Andrewes**
–	Elytral lateral border evenly curved; anterior patch round or slightly transverse, sometimes weakly zigzag	**12**
12	Median lobe of aedeagus with apical orifice smaller, opened to the left side (Fig. 37); apical lamella long and narrow, length near two times the basal width (lateral view); elytral outer apical angles usually acuminate (Fig. 21); China (west to Salween River), N. Myanmar, S. Vietnam (Fig. 59)	***P. acutidens* sp. n.**
–	Median lobe of aedeagus with apical orifice larger, opened to the left-dorsal side (Figs 38, 39); apical lamella short and wide, length a little greater than the basal width (lateral view); elytral outer apical angles usually acute or obtuse (Figs 22–25); China (east of Salween River), Myanmar, Thailand, Vietnam, Laos (Fig. 59)	**13**
13	Elytral anterior patch narrower, usually widely zigzag (Fig. 12); elytra third interval usually with two setigerous pores (the middle one absent); Taiwan	***P. ornatus formosanus* Dupuis**
–	Elytral anterior patch wider, usually nearly round (Figs 9–11); elytra third interval with three setigerous pores; other localities (Fig. 59)	***P. ornatus ornatus* Schmidt-Göbel**
14	Elytral posterior patch separate, composed of three to five small spots	**15**
–	Elytral posterior patch round, of single large spot	**17**
15	Elytral posterior patch usually obsolete on the sixth interval, if present, adjacent to the spot on the fourth and fifth intervals (Fig. 12); third interval usually with two setigerous pores only; Taiwan	***P. ornatus formosanus* Dupuis**
–	Elytral posterior patch with a small spot on the sixth interval, well separated from the larger spot on the fourth and fifth intervals (Figs 13–15); third interval always with three setigerous pores; other localities	**16**
16	Elytral yellowish patches larger, usually longer than the interval width; pronotum usually narrower (PW/PL = 1.55–1.60); lateral margins distinctly sinuate before posterior angle; median lobe of aedeagus strongly dilated (Fig. 35, total length / greatest width = 3.7); Vietnam, China (Yunnan), Myanmar, India (Andaman, Sikkim)	***P. obtusipennis* Fedorenko**
–	Elytral yellowish patches smaller, most of them equal or shorter than the interval width; pronotum usually wider (PW/PL = 1.60–1.75); lateral margins barely sinuate before posterior angle; median lobe of aedeagus a little dilated (Fig. 36, total length / greatest width = 4.8); China (Guizhou, Guangxi)	***P. obscuratus* sp. n.**
17	Larger size, 15.0–15.9 mm (largest in the subgenus); elytral posterior patch in intervals 4 to 7; elytra flat; Laos, China (Yunnan)	***P. dux* Andrewes**
–	Smaller size, 7.2–8.5 mm (smallest in the subgenus); elytral posterior patch in intervals 2 to 5 or 3 to 4; elytra distinctly convex; Myanmar (Putao), China (Mêdog)	***P. gibbosus* sp. n.**

*Pericalus aeneipennis* Louwerens and *P. distinctus* Dupuis are not included in the key. Detailed discussions on these two very little known and dubious species are provided under remarks of their similar species (*P. elegans* and *P. obtusipennis*).

### Pericalus (s. str.) gibbosus sp. n.

Taxon classificationAnimaliaColeopteraCarabidae

http://zoobank.org/58189BD2-F135-4471-A3FD-E3C34E3648E9

Figs 1 , 2 , 19 , 30 , 33 , 41 , 52 , 60

#### Type material.

**Holotype** (IZAS): male, body length = 7.2 mm, card-mounted, genitalia dissected and placed in micro vial pinned under specimen, “Myanmar, Kachin state, Putao distr., way btw. Upper Shankhaung to Wasandum; rain forest; 1075 m”; “N27.4765, E97.2060, 2016.XII.11, SHI H.L. lgt. in dead log, CAS-SEABRI exp. 2016”; “HOLOTYPE ♂ Pericalus gibbosus sp. n., des. SHI & LIANG 2018” [red label] (Fig. 19). **Paratypes** (2 ex.): 1 female (IZAS): same data as holotype. 1 female (IZAS), “Xizang, Mêdog; alt 800–1200 m, Chinese Academy of Science”; “1983.11.5–10, Han Yinheng lgt.”; “IOZ (E) 1891857”.

#### Diagnosis.

Smallest species in the subgenus, body length 7.2–8.5 mm; elytra black with four round yellowish spots, posterior patch in the third and fourth intervals, sometimes expanded onto part of the second and fifth. Pronotum cordiform, lateral margins strongly sinuate before posterior angles; disc with strong wrinkles. Elytra distinctly convex; outer apical angles rounded; sutural angles blunt; third interval with three setigerous pores; striae with large but shallow punctures. Median lobe of aedeagus with apical lamella very long, approximately one-fourth length of median lobe; endophallus with six thumb tack-like spines (Fig. 33).

#### Comparison.

This species can be readily distinguished from other species in this subgenus. The elytral pattern is similar to that of *P. dux* and *P. longicollis*, all with four round yellowish spots, but differs from both species by the elytral posterior patch in intervals 3–4, sometimes also part of 2 and 5 (versus in intervals 4–6 or 4–7); elytra much more convex; pronotum strongly sinuate before posterior angles (versus weakly or moderately sinuate); and much smaller body size.

This species is sympatric with *P. elegans* sp. n., *P. acutidens* sp. n., and *P. amplus*. It can be easily distinguished from these species by the different elytra pattern, smaller size, and very convex body.

#### Description.

Body length 7.2–8.5 mm (male holotype 7.2 mm, female paratypes 8.2–8.5 mm). *Coloration*. Dorsal surface shiny blackish, with very faint violet hue on elytra; legs, antennae, mouthparts reddish brown; elytral anterior and posterior patches as single round spots, yellow or reddish yellow; the anterior one in intervals 4–7 or 4–6, the posterior one in intervals 2–5 or 3–4 (the male holotype with elytral spots smaller than those of the two females, Figs 1–2); ventral side black. *Microsculpture* faint and isodiametric on vertex and pronotal disc, distinct and linear on elytral intervals. *Head* with very strong and regular long wrinkles all through clypeus, frons and vertex, reaching or nearly reaching level of posterior margin of eyes, 6–8 wrinkles on each side; eyes strongly prominent; temporae abruptly constricted after eyes. *Pronotum* cordiform, PW/PL = 1.33–1.38, subequal to the width of head with eyes (PW/HW = 1.00–1.05); posterior margin a little narrower than anterior margin; lateral margins rounded in the middle, strongly sinuate and then straight before posterior angles; posterior angles sharp, nearly rectangular, projecting laterally, with a seta a little before posterior angles; lateral expansions wide; disc strongly convex and wrinkled, with a pair of faint pits on each side; sub-anterior impression distinct, median line strongly incised, not reaching posterior margin; basal fovea very deep, forming trifurcate incisions, extending medially forming W-shaped sub-posterior impression. *Elytra* ovate, strongly convex; EW/EL = 0.74–0.75; much wider than pronotum, EW/PW = 1.69–1.83; apical truncation weakly curved; outer apical angles rounded, sutural angles blunt, not pointed; striae deeply incised, with large but very shallow punctures; third interval with three setigerous pores, the first one at approximately basal tenth, the second near middle, the third one close to apex; the first one adjacent to the third stria, the other two close to the second stria; all intervals distinctly convex, seventh and eighth intervals tumid apically, eighth and ninth intervals with sparse fine setae aside of umbilical series; lateral expansions slightly widened at approximately basal third. *Male genitalia* (Fig. 33) with median lobe of aedeagus slender and bent, right margin slightly sinuate near middle; apical orifice very large, opened to the left; apical lamella very long, approximately one fourth length of median lobe, flat, in ventral view length approximately 2.8 times basal width, apex rounded; endophallus with six thumb tack-like spines, arranged in two groups, three near the base of apical orifice, the other three near base of median lobe. Right paramere short, apex truncate, not extended. *Female genitalia*. Internal reproductive system (Fig. 52): bursa copulatrix with a very long lobe (inferred to accommodate the large apical lamella of aedeagus); spermatheca digitiform, with weak whorl near middle, without basal pedicel, inserted on the joint of common oviduct and bursa copulatrix; spermathecal gland long, inserted near base of spermatheca, apex strongly dilated, base forming long pedicel. Gonocoxite 2 of ovipositor (Fig. 41) scimitar-shaped, abruptly bent to the outer side at apical fifth; length approximately six times basal width; outer margin with three dorsolateral ensiform setae, the basal one finer and distant from the rest two; inner margin with one doromedial ensiform seta near apex.

#### Distribution.

Only known from Putao (North Myanmar) and Mêdog (Southeast Xizang, China). (Fig. 60)

#### Etymology.

The name *gibbosus* is Latin, meaning humped, referring to the strongly convex elytra of the new species, which are rather flat in all the other species of this subgenus.

#### Habitat.

In north Myanmar (Putao), *P. gibbosus* sp. n. was collected under bark of a large fallen log next to a path in subtropical rainforest, elevation of 1075m. This species seems to be very rare, due to only two specimens were collected, cohabitates with *P. acutidens* and *P. amplus*. *Pericalus acutidens* was collected in exactly the same microhabitat together with the new species, but it has a wider elevational range and is much more common. *Pericalus amplus* is also rare in this area.

#### Remarks.

The form of the male genitalia makes *P. gibbosus* the most unique member in the subgenus, and in the genus as well. Unlike all other examined species, *P. gibbosus* has the apical lamella very long, approximately one-fourth length of median lobe (less than one tenth in the other species); and the endophallus with thumb tack-like spines (only finely scaled in other species). This species may represent an isolated lineage in the genus; however, from the external characters, *P. gibbosus* perfectly agrees with the subgenus Pericalus. Moreover, we suspect that *Pericalus* (*s. str.*) could be paraphyletic, because all diagnostic characters for the subgenus seem to be plesiomorphic. Nevertheless, phylogeny is not the task of the present paper, and this new species is placed in *Pericalus* (*s. str.*).

### Pericalus (s. str.) dux

Taxon classificationAnimaliaColeopteraCarabidae

Andrewes, 1920

Figs 3 , 32 , 43 , 54 , 60

Andrewes 1920: 25 (type locality: Laos [Xieng Khouang]; Holotype in NHML); Csiki 1932: 1369 (catalogue); Jedlička 1963: 378.

#### Material examined.

1 female (SNU), “China, Yunnan Prov., Nabanhe N.R., Bengsaihe, Alt.700 m, 20-XI-2008, HU Jia-Yao & TANG Liang leg.”; 1 female (SNU), “Manfei, Nabanhe conv., Yunnan Prov., 10.I.2004, Li & Tang leg”.

#### Diagnosis.

Largest size of all species in the subgenus, body length 15.0–15.9 mm; dorsal surface black, elytra with four round yellowish spots; the anterior one in intervals 4–8, the posterior one in intervals 4–7. Pronotum transverse, PW/PL 1.47–1.50; lateral margins gently sinuate and then straight before nearly rectangular posterior angles; disc with fine wrinkles. Elytra flat; apex very faintly curved; outer apical angles rounded; sutural angles blunt or forming very short tooth; third interval with three setigerous pores, the middle one at approximately anterior third; striae shallow and impunctate; eighth and ninth interval with dense fine setae.

#### Comparison.

This species can be easily distinguished from all other known species in the subgenus by its larger size and distinctive elytral pattern (Fig. 3). Two other species *P. longicollis* and *P. gibbosus* also have four round spots on elytra, but *P. dux* has wider spots placed laterally. This species is sympatric with *P. ornatus* in south Yunnan. These two species can be distinguished by the differences in body sizes, the elytra patterns, and the outer apical angle of the elytra.

#### Supplemental description.

Male genitalia not studied. *Female genitalia*. Internal reproductive system (Fig. 54): spermatheca short and pedunculate, inserted on the bursa copulatrix; spermatheca tubular, approximately two times length of pedicel; pedicel short, branched near the joint; spermathecal gland absent. Gonocoxite 2 of ovipositor (Fig. 43) scimitar-shaped, gradually bent to the outer side after apical third; length approximately five times basal width; outer margin with two dorsolateral ensiform setae, the apical one placed near the middle, the basal one on the basal quarter, finer than the apical one; inner margin with one doromedial ensiform seta near apex.

#### Distribution.

Laos (Xieng Khouang, Pon Bai, Ban Sai, Muong Pek), China (Yunnan) (Fig. 60).

### Pericalus (s. str.) elegans sp. n.

Taxon classificationAnimaliaColeopteraCarabidae

http://zoobank.org/C5B8C2DF-DAD8-475A-A913-59AD66287FAB

Figs 4 , 5 , 20 , 31 , 34 , 42 , 53 , 60

#### Type material.

**Holotype** (IZAS): male, body length = 10.6 mm, board mounted, left antenna wanting, genitalia dissected and deposited in micro vial pinned under specimen, “CHINA, Xizang, Mêdog, 96km at road Bomê to Mêdog, 1413 m, N29.5837, E95.4674, 2014.VII.20, daytime, YANG X.D. lgt., 14Y0158, CCCC.” [in Chinese]; “HOLOTYPE ♂ Pericalus elegans sp. n., des. SHI & LIANG 2018” [red label] (Fig. 20). **Paratypes** (4 ex.): 1 male (IZAS), left elytra broken: “China, Tibet, Mêdog county, close to township, N29.32687 E95.32975, broadleaf forest, 1300–1500 m, 2012.VII.30D, YANG G.Y. lgt.”; “IOZ(E)1700285”. 1 female (CCCC), “CHINA, Xizang, Mêdog, Phomshen village, 1846m, N29.5767, E95.3952, 2014.VII.12, light trap, YANG X.D. lgt., 14Y0460, CCCC”. 1 male, 1 female (IZAS), “China, Tibet, Zayü county, Xia Zayü Twonship, Gadui, 28.50226, 97.00425”; “1686 m, 2001.7.8D, Liu Ye collector, Institute of Zoology”. 1 male (CRS), “Tibet - Motuo co., Hanni, VI.2013”.

#### Diagnosis.

Middle size in the subgenus, body length 10.6–11.6 mm; head and pronotum black, elytra unicolorous, cupreous green with a strong metallic hue. Pronotum strongly transverse, PW/PL = 1.65–1.80; lateral margins weakly sinuate before posterior angles; disc without transverse wrinkle. Elytra flat; outer apical angles rounded; sutural angles sharp, forming short tooth; third interval with three setigerous pores; striae shallow and impunctate. Median lobe of aedeagus evenly curved; apical orifice opened to the left side.

#### Comparison.

Only three other species in the subgenus have no yellowish pattern on the elytra. This new species can be readily distinguished from *P. cicindeloides* and *P. fascinator* by the presence of three setigerous pores on the third elytral interval (vs. four pores in the other two). From *P. aeneipennis*, which also has three setigerous pores according to the original description (further discussions, see below), this new species is distinguishable by all wrinkles long on vertex, reaching or nearly reaching level of posterior margin of eyes (vs. wrinkles short, only reaching a little beyond mid-eye level); pronotum much wider, PW/PL more than 1.65 (vs. approximately 1.30); pronotal lateral margins weakly sinuate before posterior angles (vs. strongly sinuate); posterior angles not projecting laterally (vs. projecting a little laterally); elytral outer apical angles completely rounded (vs. “hooked but not toothed”); and quite different distribution ranges.

#### Description.

Body length 10.6–11.6 mm (no significant sexual differences). *Coloration*. Head and pronotum shiny blackish, with very faint metallic hue; elytra cupreous green, with strong metallic hue; mouthparts, antennomeres 2–11 reddish brown; legs dark brown, trochanters and tarsi yellowish brown; ventral side black. *Microsculpture* faint and isodiametric on vertex and pronotal disc, distinct and isodiametric or slightly transversal on elytral intervals. *Head* with strong and irregular wrinkles; four to six short wrinkles on each side extending from clypeus to frons, middle area around frontoclypeal sulcus smooth; five to seven subparalleled long wrinkles along each side of inner margin of eye, reaching level of posterior margin of eyes; inner wrinkles forming concentric rings around the smooth vertex. Eyes very prominent; temporae gradually constricted after eyes. *Pronotum* strongly transverse, PW/PL = 1.65–1.80, subequal to width of head with eyes (PW/HW = 1.00–1.07); posterior margin a little narrower than anterior margin; lateral margins rounded in the middle, slightly sinuate before posterior angles; posterior angles a little more than rectangular angle, not projecting laterally, with a seta very close to the posterior angles; lateral expansions wide; disc weakly convex, with very faint wrinkles, with a pair of reniform shallow pits on each side; sub-anterior impression shallow, median line fine, not reaching anterior nor posterior margin; basal fovea shallow, extending medially merged with sub-posterior impression, extending posteriorly forming short oblique grooves. *Elytra* ovate, weakly convex; EW/EL = 0.73–0.78; wider than pronotum, EW/PW = 1.45–1.64; apical truncation weakly curved; outer apical angles rounded, sutural angles sharp, forming short tooth; striae shallowly incised, without punctures; third interval with three setigerous pores, the first one at approximately basal tenth, the second near middle, the third one close to apex; the first one adjacent to the third stria, the other two close to the second stria; intervals weakly convex, the eighth interval tumid apically, the eighth and ninth intervals with very sparse and fine setae aside of umbilical series; lateral expansions widely extended, strongly widened near basal third. *Male genitalia* (Fig. 34) with median lobe of aedeagus slender and bent, right margin almost straight, ventral margin evenly curved; apical orifice opened to the left; apical lamella short, a little flat, in ventral view length nearly same as basal width, in lateral view weakly constricted before apex; endophallus strongly folded, with fine scales all through length, without spines. Right paramere with apex extended and expanded, securiform. *Female genitalia*. Internal reproductive system (Fig. 53): spermatheca pedunculate, inserted on the base of common oviduct; spermatheca fusiform, longer than the pedicel; spermathecal gland inserted on the joint of spermathecal pedicel, approximately twice as long as spermatheca (including the pedicel). Gonocoxite 2 of ovipositor (Fig. 42) scimitar-shaped, abruptly bent to the outer side at apical third; length approximately six times as basal width; outer margin with three dorsolateral ensiform setae, the basal one finer than the rest two; inner margin with one doromedial ensiform seta near apex.

#### Distribution.

Only known from Mêdog and Zayü (Southeast Xizang, China). (Fig. 60).

#### Etymology.

The name *elegans* refers to the beautiful metallic color of this new species.

#### Habitat.

In southeast Tibet, this species occurs in montane rain forests, with the dominant trees being *Castanopsis* spp., *Machilus* spp., and *Elaeocarpus* spp. Elevation ranges between 1300 and 1850 m. Adults were collected on/in dead tree trunks, or attracted by light.

#### Remarks.

According to the original description (Louwerens, 1964), *P. aeneipennis* from Borneo has only three setigerous pores on the third elytral interval and seems to be very similar to the new species *P. elegans*. However, we infer that Louwerens’ species has four pores, as he either missed the last pore which is very close to elytra apex or confused it with the umbilical series, same as other species illustrated in the same paper (*P. longicollis*, *P. quadrimaculatus*. for example). Moreover, from the original description and illustration, in spite of the pores on the third elytral interval and coloration, *P. aeneipennis* is very similar to *P. cicindeloides* which is also recorded in Borneo. We suspect these two species could be conspecific. Unfortunately, no determined material of *P. aeneipennis* from Borneo was available. Therefore, these two species are retained in their present status, but *P. aeneipennis* is not included in the key to species. Nevertheless, according to those diagnostic characters mentioned above, the new species *P. elegans* is quite different from *P. aeneipennis*.

### Pericalus (s. str.) amplus

Taxon classificationAnimaliaColeopteraCarabidae

Andrewes, 1937

Figs 6 , 29 , 40 , 47 , 58 , 60

Andrewes 1937: 186 (type locality: Burma [“Ruby mines” = Mogok], holotype in NHML); Csiki 1932: 1369; Jedlička 1963: 377; Fedorenko 2017: 311 (Vietnam).

#### Material examined

(7 ex.). **China**: 1 male (IZAS), “Yunnan, Xishuangbanna, Menglun town, W. reserve station, 2004.II.13, 720 m, Wu Jie leg”. 2 females (CCCC), “Yunnan, Ruili, Bangda village, 1432 m, 2014.IX.16, night, Yang Xiaodong leg.”. **Myanmar**: 1 male, 1 female (IZAS), “Myanmar, Kachin state, Putao distr., 5km NW. of Upper Shankhaung; rain forest; 666 m, N27.4415, E97.2584, 2016.XII.21, SHI H.L. lgt., in dead log; CAS-SEABRI exp. 2016”. 1 female (IZAS), “Myanmar, Kachin state, Putao distr., way btw. Upper Shankhaung to Wasandum; rain forest; 1075 m, N27.4765, E97.2060, 2016.XII.11, SHI H.L. lgt., in dead log; CAS-SEABRI exp. 2016”. **Vietnam**: 1 male (IZAS), “TONKIN, Hoa-Binh, leg., A de Cooman”.

#### Diagnosis.

Medium size in the subgenus, body length 9.4–10.0 mm; dorsal surface black, elytra without metallic hue; anterior patch zigzag, five intervals wide, in the third to seventh intervals; posterior patch separate, composed of four small spots, in intervals 2–3, 4–5, 6, and 7–8 respectively. Pronotum transverse, PW/PL 1.56–1.65; lateral margins strongly sinuate before posterior angles. Elytral apex slightly curved, outer apical angles rounded; sutural angles blunt; third interval with four setigerous pores, the middle two at approximately anterior two-fifth and two-third; ninth interval with sparse fine setae. Median lobe of aedeagus strongly sinuate on ventral margin.

#### Comparison.

Only three species in the subgenus have four setigerous pores in the third interval of the elytra, but the other two species *P. cicindeloides* and *P. fascinator* have no yellowish patches on the elytra. This species is very similar to *P. obtusipennis* in elytral pattern. For more detailed comparisons between them, see remarks of the latter species (p. 38).

#### Supplemental description.

*Male genitalia* (Fig. 40). Median lobe of aedeagus fine, abruptly bent after base, forming a distinct angle on the ventral margin; ventral margin strongly sinuate near middle, dorsal margin concaved near base; apical orifice opened to the left; apical lamella small, a little flat, gradually narrowed to apex, slightly bent dorsally in lateral view; endophallus with fine scales only on basal half, without spines. *Female genitalia*. Internal reproductive system (Fig. 58): spermatheca pedunculate, inserted on the joint of common oviduct and bursa copulatrix; spermathecal body fusiform, apex pointed, longer than the pedicel, distinctly bent; spermathecal gland inserted on the joint of spermathecal pedicel, apex shortly dilated, approximately twice as long as spermatheca. Gonocoxite 2 of ovipositor (Fig. 47) scimitar-shaped, abruptly bent to the outer side at apical fifth; length approximately five times basal width; outer margin with three dorsolateral ensiform setae, the basal one finer than and a little distant from the other two; one doromedial ensiform seta near apex.

#### Distribution.

China (Yunnan), Myanmar (Mogok, Putao), India (Assam), N Vietnam. This species is sympatric with *P. acutidens* sp. n. in north Myanmar and Yunnan, but much rarer. (Fig. 60)

### Pericalus (s. str.) acutidens sp. n.

Taxon classificationAnimaliaColeopteraCarabidae

http://zoobank.org/C260316D-8B32-4B34-A740-6D50226B1816

Figs 7 , 8 , 17 , 21 , 37 , 45 , 56 , 59

Pericalus formosanus (in part); Fedorenko 2017: 311.

#### Type material.

**Holotype** (IZAS): male, body length = 10.4 mm, board mounted, genitalia dissected and deposited in micro vial pinned under specimen, “Yunnan, Longchuan county, Mangdong, 1770 m, 2016.X.2, night, Yang Xiaodong leg., 16Y, CCCC” [in Chinese]; “HOLOTYPE ♂ Pericalus acutidens sp. n., des. SHI & LIANG 2018” [red label] (Fig. 17). **Paratypes** (36 ex.): **Yunnan**: 1 male, 2 females (CCCC), same data as holotype. 2 males, 1 female (CCCC), same data as holotype, but date 2016.IX.28. 4 males, 1 female (IZAS), same data as holotype, but date 2016.IX.30. 1 male, 3 females (CCCC), same data as holotype, but date 2016.VI.3. 1 female (CCCC), same data as holotype, but date 2016.IX.27, beating on vegetation. 1 female (IZAS), “Yunnan, Gongshan county, Dulongjiang, Maku, 1540m, 2015.VII.23, Yang Xiaodong leg”. 1 female (IZAS), “Yunnan Prov., Longyang, Mangkuan, Baihualing, on shrub, 25.30985, 98.79485, 1440 m, 2007.10.10 day, David Kavanaugh coll”. 1 female (IZAS), “Yingjiang county, Xima, Huihe power station, 1514 m, board leaf forest, 2013.IX.20, Yang Xiaodong leg”. 1 female (IZAS), “Yunnan, Nabang, 2013.IX.7 Zhu Xiaoyu leg”. 2 males (IZAS), “Yunnan, Ruili, Bangda village, 1432 m, 2014.IX.14, light trap, Yang Xiaodong leg”. 3 males, 1 female (CCCC), “Yunnan, Ruili, Bangda village, 1432m, 2014.IX.16, night, Yang Xiaodong leg”. 1 female (IZAS), “Yunnan, Ruili, Bangda Mt., 2014.IX.14, light trap, Cai Yinan leg”. 1 female (IZAS), “Yunnan, Ruili, Bangda Mt., 1450m, 2015.VIII.30, mixed forest, night, Lu Yanquan leg”. **Xizang**: 1 male (IZAS), “Xizang, Medog county, 850–900 m, 1987.II.19, Lin Zai leg”. **Myanmar**: 4 males, 1 female (IZAS), “Myanmar, Kachin state, Putao distr., way btw. Upper Shankhaung to Wasandum; rain forest; 1075 m, N27.4765, E97.2060, 2016.XII.11, SHI H.L. lgt., in dead log; CAS-SEABRI exp. 2016”. 1 male (IZAS), “Myanmar, Kachin state, Putao distr., 5 km NW. of Upper Shankhaung; rain forest; 666 m, N27.4415, E97.2584, 2016.XII.21, SHI H.L. lgt., in dead log; CAS-SEABRI exp. 2016”. 1 male (IZAS), “Myanmar, Kachin state, Putao distr., way btw. Ziradum and camp I; rain forest; in dead log; 27.5679, 97.1062 1022 m – 27.5991, 96.9948 1593 m, 2016.XII.14, SHI H.L. lgt. CAS-SEABRI exp. 2016”.

#### Diagnosis.

Medium size in the subgenus, body length 9.2–12.0 mm; dorsal surface black, elytra usually with faint cyan hue; anterior patch round in form, usually slightly transverse and zigzag, three to five intervals wide; posterior patch separate, composed of three small spots, normally on intervals 2–3, 4–5, and 7 respectively. Pronotum lateral margins sinuate before posterior angles, posterior angles nearly rectangular. Elytra plain; apical truncation distinctly curved; outer apical angles acuminate, forming sharp tooth (Fig. 21); sutural angles blunt; third interval with three setigerous pores. Median lobe of aedeagus with apical orifice small, opened to the left side; apical lamella narrow and long, length near two times the basal width.

#### Comparison.

The new species is very similar and close to *P. ornatus*, but can be distinguished by male genital characters: (1) in *P. acutidens*, the apical orifice of aedeagus is much smaller and opened exactly to the left side of median lobe; larger and opened to the left-dorsal side in *P. ornatus*; (2) in lateral view, median lobe of aedeagus a little narrower and less bent in the new species than in *P. ornatus*; (3) the apical lamella is a little narrower and longer in *P. acutidens*; (4) in ventral view, apical lamella of *P. acutidens* is almost straightly pointed to apex; in *P. ornatus*, the apical lamella is slightly bent to the right.

Externally, these two species are different in: elytral outer apical angles generally acuminate, projected outwards, forming short tooth in *P. acutidens* (Fig. 21); not or less pointed, acute, obtuse, or rarely rounded in *P. ornatus* (Figs 22–26). *P. acutidens* is also different from *P. o. ornatus* in elytral anterior patch usually a little transverse (versus usually nearly round), and from *P. o. formosanus* in elytral third interval with three setigerous pores (versus usually two pores). Generally, the difference on elytral outer apical angles well distinguishes these two species for specimens from China. But in S. Vietnam, both species have the outer apical angles acuminate or acute (Figs 1–4, 6–7 in Fedorenko, 2017). Thus the differentiation of them through external characters can be difficult sometimes. For specimens from China, it is easier to diagnose *P. acutidens* n. sp. from *P. ornatus* by their allopatric distributions (Fig. 59), but they are sympatric in at least S. Vietnam.

The new species is sympatric with *P. obtusipennis* and *P. amplus* in SW. Yunnan, and they all have similar elytral patterns. From the latter two, *P. acutidens* can be easily distinguished by the acuminate elytral apical outer angles and the elytral posterior patches which do not have a separate small spot on the sixth interval.

#### Description.

Body length 9.2–12.0 mm. *Coloration*. Dorsal surface black, elytra with faint cyan metallic hue, with yellowish patches; mouthparts, antennomeres 2–11 reddish brown; legs blackish, tarsus reddish brown; ventral side black. Elytral anterior patch round in form, generally a little transverse and zigzag, in the fourth to sixth intervals, sometimes also in parts of the third and seventh. Elytral posterior patch divided into three small spots, usually in intervals 2–3, 4–5, and 7 respectively; occasionally the first one only on the third interval, the second one only on the fifth interval or on the fourth, fifth, and sixth intervals. *Microsculpture* faint and nearly isodiametric on vertex and pronotal disc, strong and linear on elytral intervals. *Head* densely wrinkled; three or four wrinkles on each side extending from clypeus to frons; seven to ten fine wrinkles along each side of inner margin of eye, reaching level of posterior margins of eyes; wrinkles very weak on vertex, occiput almost smooth. Eyes strongly prominent; temporae gradually constricted after eyes. *Pronotum* strongly transverse, PW/PL = 1.52–1.61, subequal to the width of head with eyes (PW/HW = 0.96–1.02); posterior margin subequal to the width of anterior margin; lateral margins rounded in the middle, distinctly sinuate before posterior angles; posterior angles nearly rectangular, apex sharp, not projecting laterally; lateral expansions wide and rugose; disc a little convex, with fine wrinkles, with a pair of shallow pits on each side; sub-anterior impression barely visible, median line fine, not reaching anterior nor posterior margin; basal fovea shallow, extending medially merged with the shallow sub-posterior impression, extended posteriorly forming very shallow short oblique grooves. *Elytra* ovate, weakly convex; EW/EL = 0.65–0.73; much wider than pronotum, EW/PW = 1.50–1.65; apical truncation distinctly curved; outer apical angles generally acuminate, or rarely acute; sutural angles blunt; striae moderately incised, without punctures; third interval with three setigerous pores, the first one at approximately basal eighth, the second one at approximately middle, the third one close to apex; the first one adjacent to the third stria, the other two close to the second stria; intervals slightly convex, the eighth interval tumid apically, the eighth and ninth interval with sparse fine setae aside of umbilical series; lateral expansions narrowly extended, a little widened near basal third. *Male genitalia* (Fig. 37). Median lobe of aedeagus slender and bent, weakly sinuate in ventral and dorsal view; in lateral view, ventral margin evenly curved in the middle; apical orifice small, opened to the left; apical lamella narrow and long, sides parallel in ventral view, in lateral view gradually narrowed to apex, length near two times the basal width, apex rounded; endophallus simple, with very fine scales all through length, without spines. Right paramere with apex extended and expanded, round in form. *Female genitalia*. Internal reproductive system (Fig. 56): spermatheca pedunculate, inserted on the base of common oviduct; spermathecal body fusiform, longer than the pedicel, distinctly bent; spermathecal gland inserted on the joint of spermathecal pedicel, apex not dilated (probably spermathecal gland apex missing), close to the length of spermatheca. Gonocoxite 2 of ovipositor (Fig. 45) scimitar-shaped, abruptly bent to the outer side at apical third; length approximately six times basal width; outer margin with three or four dorsolateral ensiform setae, the basal one finer than the other ones; inner margin with one doromedial ensiform seta near apex.

#### Distribution.

A relatively widely distributed species, known from several localities west to Salween River: Myanmar: Putao, Shan States; China: Xizang (Mêdog), Yunnan (Nujiang, Dehong, Baoshan Prefectures), S. Vietnam (Fedorenko 2017); probably also in north India, Thailand, and Cambodia (Fig. 59).

#### Etymology.

The name *acutidens* comes from Latin, referring to the generally acuminate elytral outer apical angles of the new species.

#### Habitat.

According to the collecting data, this new species prefers tropical forests with the elevational range between 1000m and 1500m. Adults were collected on/in dead tree trunks, or attracted by light at night.

#### Remarks.

This new species was recorded by Fedorenko (2017) as *P. formosanus* in south Vietnam. Based on the figures of male genitalia in Fedorenko (2017), we determined that his Vietnam *P. formosanus* is not the true *P. formosanus* of Dupuis, but is exactly identical to our new species.

From the examined materials in the present study, the new species is strictly allopatric with *P. ornatus* in China. The natural boundary in China between them seems to be the Salween River (Fig. 59). But we didn’t examine any material form regions between the Salween River and Mekong River. These two species are sympatric in south Vietnam, and the new species is rarer than *P. ornatus* (Fedorenko, 2017).

*Pericalus ornatus* was previously recorded from North India (Assam, Sikkim, Garo Hills), Myanmar (North Shan States, Karin Chebà), Thailand, Laos, and Cambodia. We doubt some of these records are actually *P. acutidens* sp. n. The record from North Shan States (Andrewes 1923) is very close to Ruili (Yunnan) where *P. acutidens* sp. n. was recorded and *P. ornatus* does not occur. Thus this record should be confirmed to *P. acutidens* sp. n. According to the confirmed records (solid spots in Fig. 59) we inferred that these two species could be sympatric in S. Laos, S. Myanmar, Thailand, and Cambodia, and *P. ornatus* may not occur in N. India and N. Myanmar. The records from north India (Andrewes 1923) may refer to *P. acutidens* sp. n. or some other unknown species (empty red circles in Fig. 59).

### Pericalus (s. str.) ornatus ornatus

Taxon classificationAnimaliaColeopteraCarabidae

Schmidt-Göbel, 1846

Figs 9 , 10 , 11 , 16 , 22 , 23 , 24 , 38 , 48 , 51 , 59

Schmidt-Göbel 1846: 86 (type locality: Burma [Tenasserim]; syntypes in NMPC); Bates 1892: 411 (Karin Chebà); Dupuis 1913: 83; Andrewes 1923: 49 (India, Thailand, Laos, Cambodia); Csiki 1932: 1369 (catalogue); Andrewes 1937: 185; Jedlička 1963: 379 (Vietnam); Fedorenko 2017: 311.

#### Material examined

**Figures 16–20.**
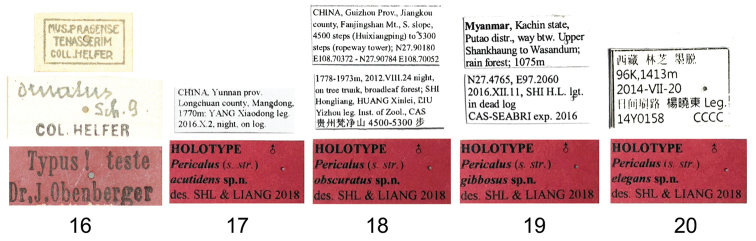
Labels of types: **16** *P. ornatus ornatus* Schmidt-Göbel, syntype (NMPC) **17** *P. acutidens* sp. n., holotype (IZAS) **18** *P. obscuratus* sp. n., holotype (IZAS) **19** *P. gibbosus* sp. n., holotype (IZAS) **20** *P. elegans* sp. n., holotype (IZAS);

**Figures 21–32.**
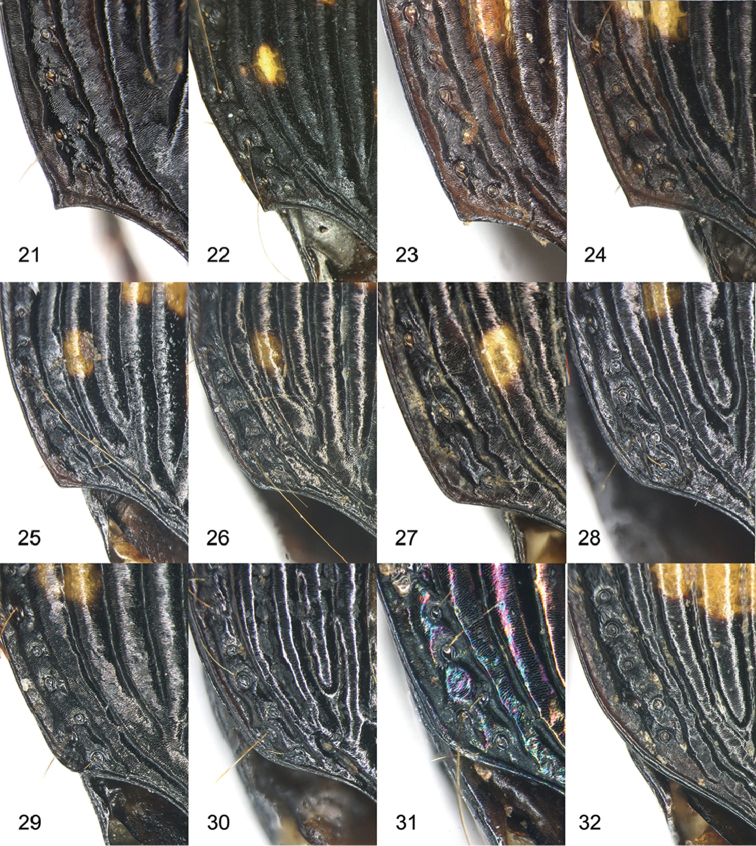
Elytral outer apical angle (left): **21** *P. acutidens* sp. n., Yunnan (Ruli) **22** *P. ornatus ornatus* Schmidt-Göbel, Yunnan (Mengla) **23** *P. ornatus ornatus* Schmidt-Göbel, Vietnam (Tonkin) **24** *P. ornatus ornatus* Schmidt-Göbel, Hainan (Baisha) **25** *P. ornatus formosanus* Dupuis, Taiwan (Miaoli) **26** *P. ornatus formosanus* Dupuis, Taiwan (Nantou) **27** *P. obtusipennis* Fedorenko, Yunnan (Pianma) **28** *P. obscuratus* sp. n., Guizhou (Fanjingshan) **29** *P. amplus* Andrewes, Myanmar (Putao) **30** *P. gibbosus* sp. n., Myanmar (Putao) **31** *P. elegans* sp. n., Xizang (Mêdog) **32** *P. dux* Andrewes, Yunnan (Jinghong).

**Figures 33–34.**
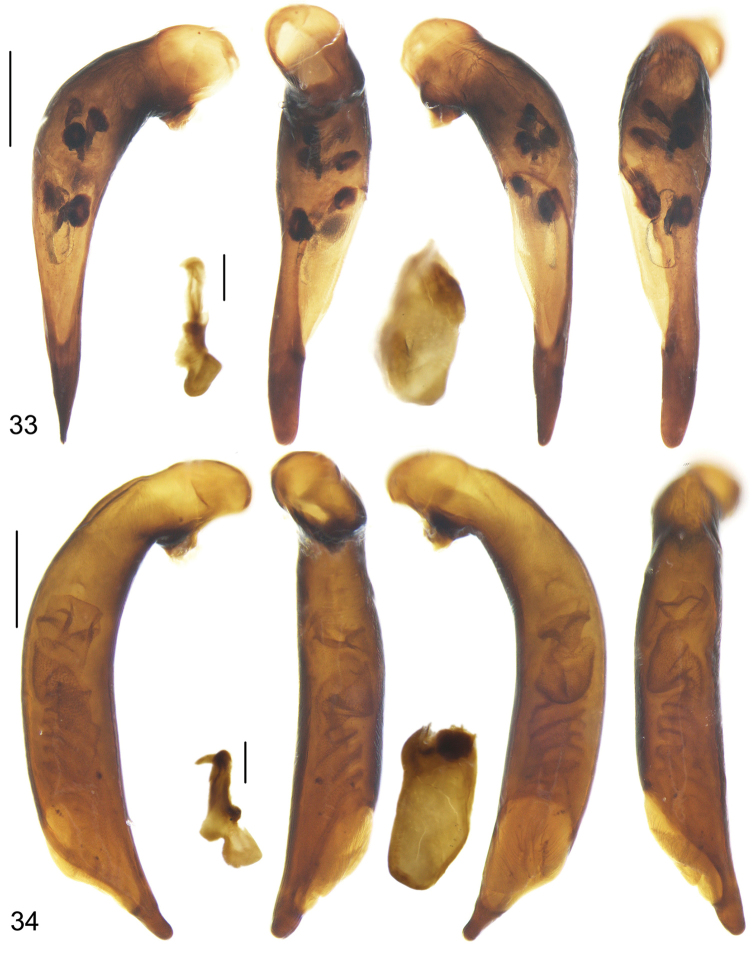
Aedeagus: right-lateral, ventral, left-lateral, and dorsal views of median lobe, scale bars 0.5 mm. Right and left parameres, scale bars 0.2 mm. **33** *P. gibbosus* sp. n., holotype, Myanmar (Putao) **34** *P. elegans* sp. n., holotype, Xizang (Mêdog).

(101 ex.). Syntype of *Pericalus ornatus*, 1 male (NMPC), “MUS. PRAGENSE TENASSERIM COLL. HELFER”; “*ornatus Sch.g.* COL. HELFER”; “Typus! Teste Dr. J.Obenberger” [red label] (Fig. 16). **Yunnan**: 5 ex (IZAS), “Yunnan, Jinghong, Ganlanba, 650 m, Wang Shuyong / Zang Lingchao leg., 1957.III.16”. 1 male (IZAS), “Yunnan, Jinghong, Ganmanta, 580 m, Pu Fuji leg., 1957.IV.22”. 1 male (IZAS), “Yunnan, Mengla county, Menglun town, W. reserve station, 2004.II.12, 720 m, Wu Jie leg”. 1 female (IZAS), “Yunnan, Mengla county, Menglun town, W. reserve station, 2004.II.09, 560 m, Wu Jie leg”. 1 male, 1 female (IZAS), “”Yunnan, Menglun, Xishuangbanna tropical Botany Garden, 2009.IX.10, Zhu Xiaoyu leg.”. 1 male (IZAS), “Yunnan, Mengla county, Wangtianshu, 2004.II.15, Wu Jie leg., 730 m”. 1 male (IZAS), “Yunnan, Mengla county, Bubang, 2009.V.11, Li Hu leg”. 2 females (IZAS), “Yunnan, Mengla county, 55km in Menglun town, 703 m, 2013.X.3, Yang Xiaodong leg”. 1 male (IZAS), “Yunnan, Mengla county, 2999 km at G213 road, 2012.IX.22, Yang Xiaodong leg”. 12 ex (IZAS), “China, Yunnan, Mengla, Biodiversity Corridor, N21.40482 E101.63035, 660m, Liang Hongbin & Li Kaiqian leg., 2011.IV.25D”. 1 male (IZAS), “China, Yunnan, Mengla, Nanping, 765m, N27 17.206’, E101 23.631’, 2009.V.12, Li & Yang”. 1 male (CCCC), “Yunnan, Hekou county, Huayudong, 150 m, 2010.IV.26 N, Lin Wensin leg”. 37 ex (IZAS), “Yunnan, Jinping county, Mengla, 400 m, Huang Keren et. leg., 1956.IV.25”. **Hainan**: 1 male 2 females (IZAS) “Hainan Prov., Yinggeling Nat. Res., Baisha, Hongxin, N19.07495, E109.52198, 429m, 2008.11.16, Shi H.L. coll. on dead log”. 1 female (IZAS), “Hainan, Ledong, Jianfengling, Mingfenggu, N18.74393, E108.84453, 950 m, 2012.IV.20N, Shi H.L. Liu Y. leg”. 1 male (IZAS), “Hainan, Ledong county, Jianfengling, Tianchi, 950 m, 2011.V.20–23, Lin Wensin”. **Vietnam**: 29 ex (IZAS), “TONKIN, Hoa-Binh, leg. A de Cooman”.

#### Diagnosis.

Medium size in the subgenus, body length 8.4–12.5 mm; dorsal surface black, elytra usually with faint cyan hue; anterior patch usually nearly round, three to five intervals wide; posterior patch separate, composed of three small spots, in intervals 2–3, 4–6, and 7 respectively, the middle one variable, when narrowest, present on the fifth interval only. Pronotum transverse, PW/PL 1.53–1.63; lateral margins sinuate before posterior angles, posterior angles nearly rectangular; disc with fine wrinkles. Elytra flat; apical truncation distinctly curved; outer apical angles usually acute (Figs 22, 24), sometimes obtuse (Fig. 23) or acuminate (in S. Vietnam); sutural angles blunt; third interval with three setigerous pores, the second one near middle of elytron. Median lobe of aedeagus with apical orifice large, opened to left-dorsal side (Fig. 38).

#### Comparison.

*P. o. ornatus* is distinguishable from similar taxa by the acute or obtuse elytral outer apical angles and elytral anterior patch usually round. *P. o. ornatus* is sympatric with *P. obtusipennis* and *P. amplus* in China, and all have similar elytral pattern. From the latter two species, *P. ornatus* can be easily distinguished by the elytral apical outer angles not rounded and without separate spot on the sixth interval.

#### Supplemental description.

*Male genitalia* (Fig. 38). Median lobe of aedeagus slender and bent; in lateral view, ventral margin evenly curved in the middle; in ventral view, right margin barely sinuate near middle, apical lamella slightly bent to the right; apical orifice larger than *P. acutidens*, opened to left-dorsal side; apical lamella small, side paralleled in ventral view, in lateral view gradually narrowed to apex, length a little greater than the basal width, apex rounded; endophallus with very fine scales on some regions, without spines. *Female genitalia*. Internal reproductive system (Fig. 51): spermatheca pedunculate, inserted on the joint of common oviduct and bursa copulatrix; spermathecal body fusiform, a little longer than the pedicel, distinctly bent; spermathecal gland inserted on the joint of spermathecal pedicel, apex dilated, much longer than spermatheca. Gonocoxite 2 of ovipositor (Fig. 48) scimitar-shaped, abruptly bent to the outer side at apical fourth; length approximately six times as basal width; outer margin with three dorsolateral ensiform setae, the basal one a little distant from the other two; inner margin with one doromedial ensiform seta near apex.

#### Distribution.

A relatively widely distributed species, known from localities east to Salween River: China: Yunnan (Xishuangbanna and Honghe Prefectures), Hainan; Myanmar (Tenasserim), Thailand, Vietnam, Laos, Cambodia (Fig. 59).

#### Remarks.

We attribute above specimens to *P. ornatus* based on the descriptions (Schmidt-Göbel 1846; Andrewes 1923) and a photograph of one syntype we took in NMPC years ago (Figs 9, 16). The syntype of *P. ornatus* has an almost round elytral anterior patch and the outer apical angles acute, not acuminate. It is agrees perfectly with the examined materials from southeast Yunnan and Tonkin. They are quite probably the same species, although the genitalia of syntype were not studied.

The specimens from Hainan are distinctive in having a slightly different elytral pattern: anterior patch generally smaller, usually three or four intervals wide (Fig. 11); specimens from other localities (Yunnan, Tonkin, and Myanmar) have the anterior patch generally larger, usually five intervals wide (Figs 9, 10). But all examined specimens of *P. ornatus ornatus* have the anterior patch nearly rounded (length subequal to the width).

### Pericalus (s. str.) ornatus formosanus

Taxon classificationAnimaliaColeopteraCarabidae

Dupuis, 1913

Figs 12 , 25 , 26 , 39 , 46 , 57 , 59

Dupuis 1913: 83 (type locality: Taiwan [Moozan, Sokutsu]; syntypes in IRSN); Jedlička 1963: 379; Kabak 2003: 437 (catalogue).

#### Type localities.

“Moozan” is a misspelling of Hoozan (sometimes also Hozan), referring to Fengshan (N22.61, E120.35) in Kaohsiung county, south Taiwan. Sokutsu refers to Hsiaolin (N23.16, E120.64) in Kaohsiung county.

#### Material examined

(13 ex.). 1 male (CCCC), “Taiwan, Pingtung County, Chunri, Dahanshan, 2009.V.3 D”. 1 male, 2 females (CCCC), “Taiwan, Kaohsiung County, Hsiaoguanshan, 1996.X.12, Chou Wen-I leg”. 1 female (CCCC), “Taiwan, Kaohsiung, Tengjhih, 2008.V.24 N”. 2 males, 2 females (CCCC), “Taiwan, Nantou county, Xitou, 1995.III.25”. 1 male, 1 female (CCCC), “Taiwan, Miaoli county, Sanyi, Guandaoshan, 1995.VI.2”. 1 female (CCCC), “Taiwan, Yilan county, Datong, Renze, 1998.IV.11”. 1 female (CCCC), “Taiwan, Taichung county, Heping, Anmashan”.

#### Diagnosis.

Medium size species, body length 9.5–10.5 mm; dorsal surface black, elytra usually with faint cyan hue; anterior patch transverse, a little zigzag (more transverse and zigzag than *P. o. ornatus*), usually in the fourth to sixth intervals; posterior patch separate, composed of three small spots, in intervals 2–3, 4–5, and 7 respectively. Pronotum transverse, PW/PL 1.50–1.58; lateral margins sinuate before posterior angles, posterior angles nearly rectangular; disc with fine wrinkles. Elytra flat; apical truncation distinctly curved; outer apical angles usually obtuse (Fig. 25), occasionally rounded (Fig. 26) or acute; sutural angles blunt; third interval usually with two setigerous pores (occasionally three as other species), the middle one usually missing. Median lobe of aedeagus with apical orifice opened to left-dorsal side (Fig. 39).

#### Comparison.

This subspecies is different from the nominate subspecies in: (1) elytral anterior patch generally more or less transverse (width a little more than length), and somewhat zigzag in *P. o. formosanus* (Fig. 12); nearly round (width subequal to the length) and not zigzag in *P. o. ornatus* (Figs 9–11). (2) *P. o. formosanus* generally with only two setigerous pores on the third elytral interval (occasionally three); in *P. o. ornatus* always with three setigerous pores; (3) in *P. o. formosanus*, the elytral outer apical angles obtuse in most examined specimens (Fig. 25); in *P. o. ornatus* usually acute (Figs 22, 24). But for some individuals, the shape of elytral outer apical angles cannot differentiate these two subspecies. The male genitalia of these two subspecies have no significant differences (Figs 38, 39).

*Pericalus o. formosanus* is very similar with *P. acutidens* in elytra pattern, but can be distinguished by two pores on elytral third interval, different male genitalia, and distribution. This subspecies is endemic and the only *Pericalus* species in Taiwan.

#### Distribution.

Only known from Taiwan (Fig. 59).

#### Remarks.

According to our examined specimens, the male genitalia of *P. formosanus* have no significant differences from those of *P. ornatus* from the Asian continent. Considering the external differences between *P. ornatus* as well as their allopatric distributions, it is preferable to assign *P. formosanus* as a subspecies of *P. ornatus* rather than synonymize them. Fedorenko (2017) recorded *P. formosanus* from S. Vietnam based on a misidentification of *P. acutidens* sp. n.

In most examined specimens have only two setigerous pores in the elytral third interval (the middle one missing), except for two specimens with three pores on right elytron only (Fig. 12). We consider the presence of the middle pore as individual aberration, and consider that the number of pores in third elytral interval is a good taxonomical character to distinguish between these two subspecies. For the elytral outer apical angles, most examined specimens have an obtuse angle (Fig. 25), but it is rounded in one specimen from Nantou (Fig. 26), and acute in one specimen from Kaohsiung and two of the five photographed syntypes (http://digitmuse.nmns.edu.tw).

### Pericalus (s. str.) obtusipennis

Taxon classificationAnimaliaColeopteraCarabidae

Fedorenko, 2017

**Figures 35–36.**
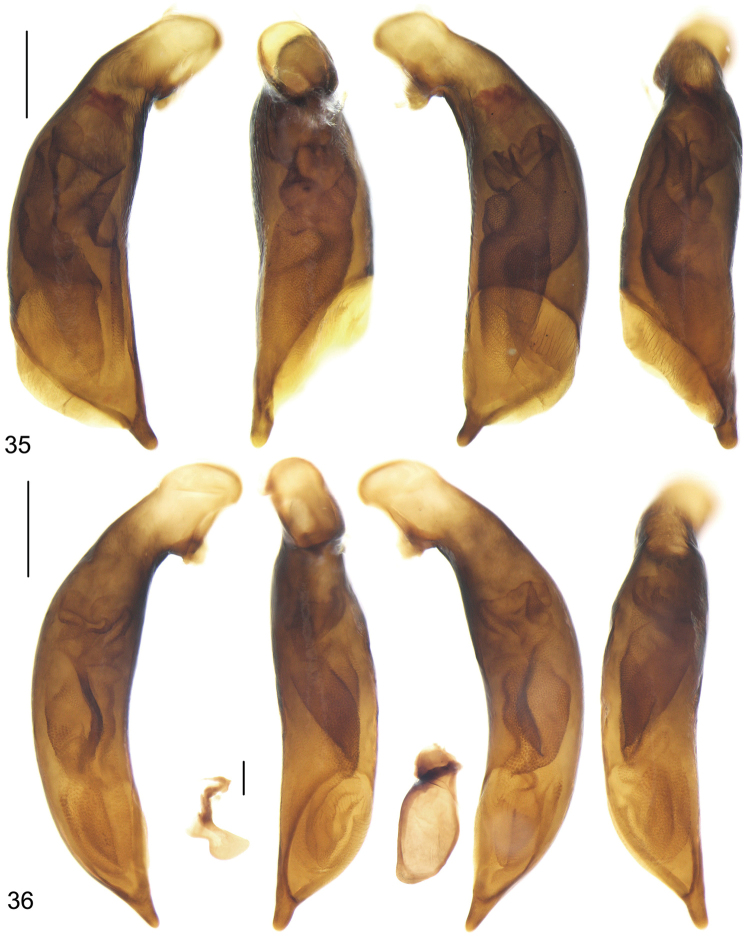
Aedeagus: right-lateral, ventral, left-lateral, and dorsal views of median lobe, scale bars 0.5 mm. Right and left parameres, scale bar 0.2 mm. **35** *P. obtusipennis* Fedorenko, Yunnan (Pianma) **36** *P. obscuratus* sp. n., holotype, Guizhou (Fanjingshan).

**Figures 37–38.**
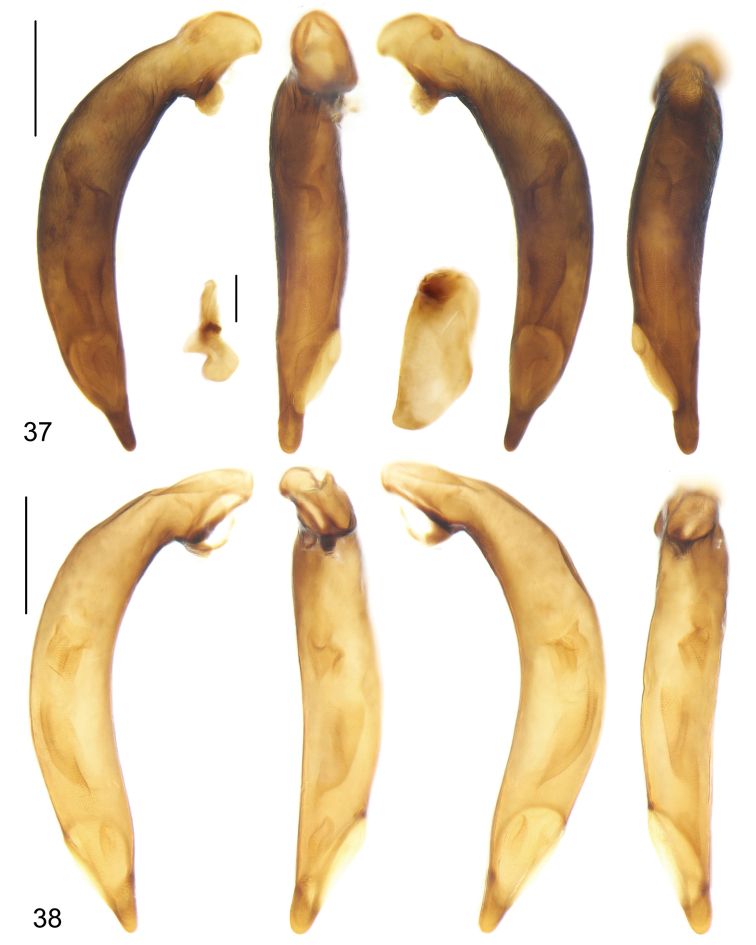
Aedegus: right-lateral, ventral, left-lateral, and dorsal views of median lobe, scale bars 0.5 mm. Right and left parameres, scale bar 0.2 mm. **37** *P. acutidens* sp. n. Holotype, Yunnan (Longchuan) **38** *P. ornatus ornatus* Schmidt-Göbel, Yunnan (Mengla).

**Figures 39–40.**
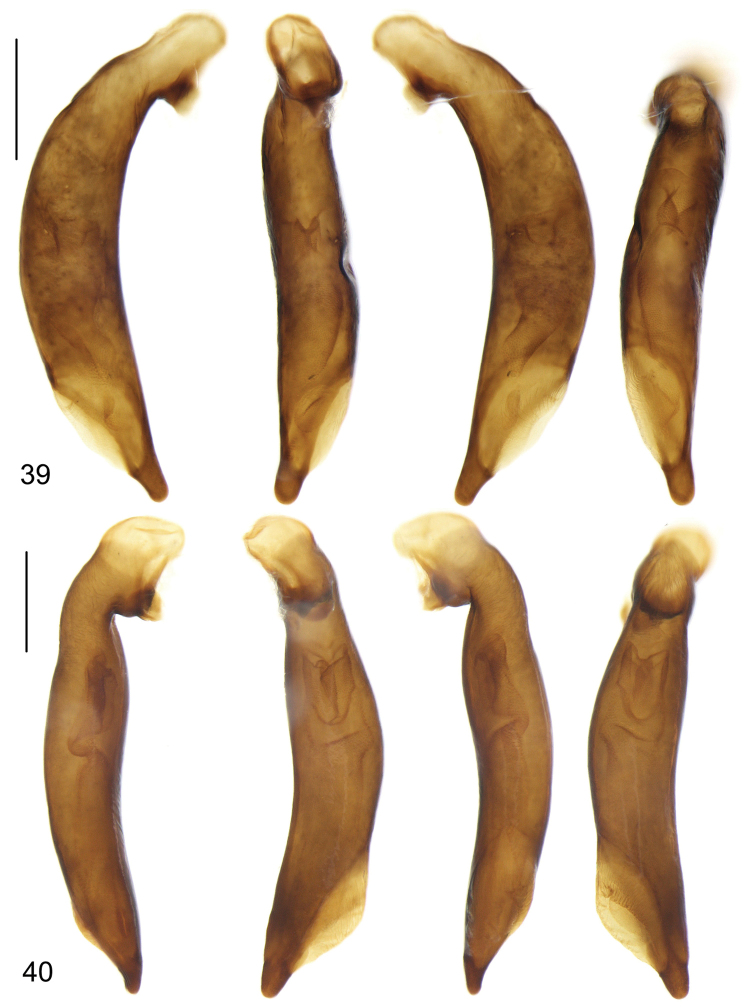
Median lobe of aedeagus, right-lateral, ventral, left-lateral, dorsal views **39** *P. ornatus formosanus* Dupuis, Taiwan (Miaoli) **40** *P. amplus* Andrewes, Myanmar (Putao). Scale bars: 0.5 mm.

**Figures 41–51.**
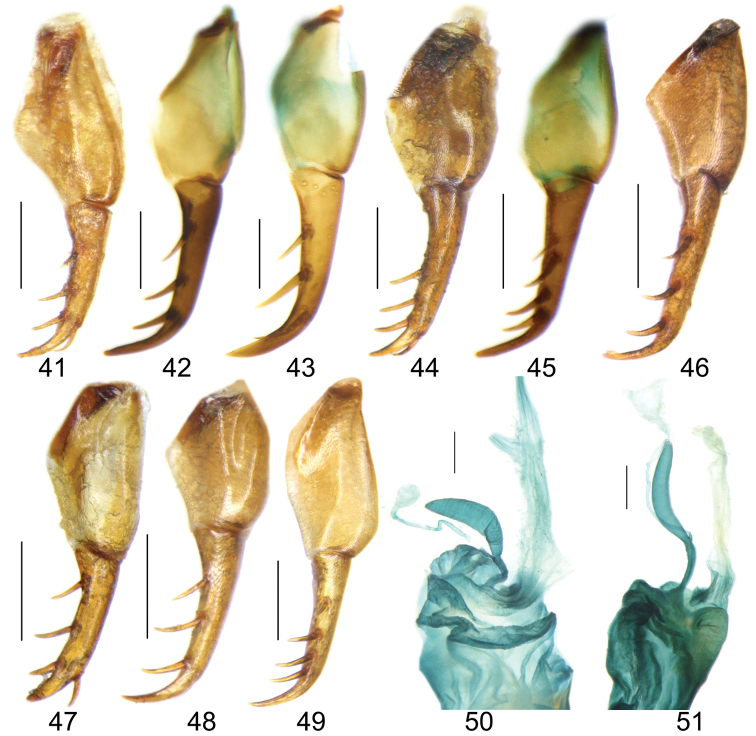
**41–49** Stylomere of right ovipositor, ventral view, **41** *P. gibbosus* sp. n., Xizang (Mêdog) **42** *P. elegans* sp. n., Xizang (Mêdog) **43** *P. dux* Andrewes, Yunnan (Jinghong) **44** *P. obscuratus* sp. n., Guizhou (Fanjingshan) **45** *P. acutidens* sp. n., Yunnan (Longchuan) **46** *P. ornatus formosanus* Dupuis, Taiwan (Miaoli) **47** *P. amplus* Andrewes, Myanmar (Putao) **48** *P. ornatus ornatus* Schmidt-Göbel, Hainan (Baisha) **49** *P. obtusipennis* Fedorenko, Yunnan (Longling) **50–51** Female reproductive system **50** *P. obtusipennis* Fedorenko, Yunnan (Longling) **51** *P. ornatus ornatus* Schmidt-Göbel, Yunnan (Mengla). Scale bars: 0.2 mm.

**Figures 52–58.**
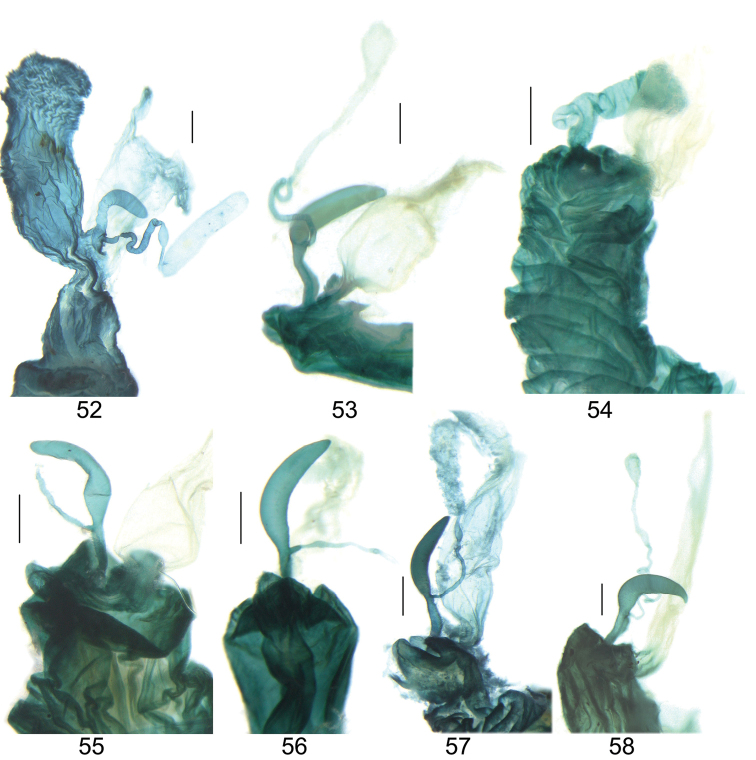
Female reproductive system, **52** *P. gibbosus* sp. n., Xizang (Mêdog) **53** *P. elegans* sp. n., Xizang (Mêdog) **54** *P. dux* Andrewes, Yunnan (Jinghong) **55** *P. obscuratus* sp. n., paratype, Guizhou (Fanjingshan) **56** *P. acutidens* sp. n., Yunnan (Longchuan) **57** *P. ornatus formosanus* Dupuis, Taiwan (Yilan) **58** *P. amplus* Andrewes, Myanmar (Putao). Scale bars: 0.2 mm.

**Figure 59.**
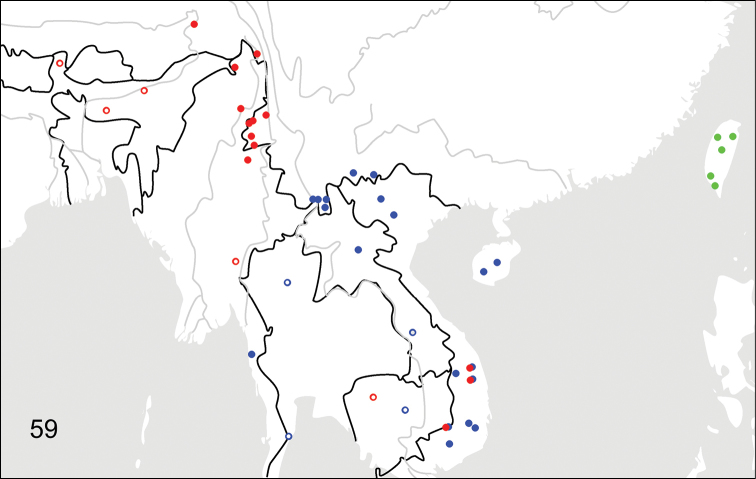
Distribution map for *P. acutidens* sp. n. (red); *P. ornatus ornatus* Schmidt-Göbel (blue); *P. ornatus formosanus* Dupuis (green); dubious records marked by hollow spots.

**Figure 60.**
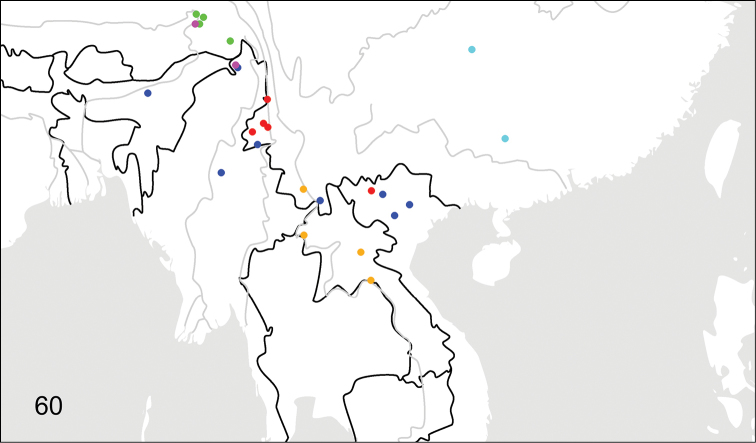
Distribution map for *P. obtusipennis* Fedorenko (red); *P. obscuratus* sp. n. (cyan); *P. amplus* Andrewes (blue); *P. gibbosus* sp. n. (magenta); *P. elegans* sp. n. (green); *P. dux* Andrewes (orange).

Figs 15 , 27 , 35 , 49 , 50 , 60

Fedorenko 2017: 308 (type locality: Vietnam [Lao Cai]; holotype in ZMMU).

#### Material examined

(7 ex.). 1 male (IZAS), “China, Yunnan prov., Nujiang, Lushui county, Pianma town, Gangfang vill., Xiaobadi; 2051 m, 2015.VI.9, light trap, Yang X.D. lgt.”; 2 males (IZAS), “China, Yunnan prov., Lushui county, Pianma township, 1900m”. 1 male (IZAS), “CHINA, Yunnan Prov., Lushui Co., Pianma, Ganheluo, riverside, 26.06210N, 98.61966E, 2100 m, 2005.V.13 day, Liang H.B. collector”. 1 male (IZAS), “CHINA, Yunnan Prov., Tengchong Co., Wuhe, Zhengding Forest station, 24.85458N, 98.73743E”; 1828 m, 2005.V.26 N, Liang H.B. collector”. 1 female (IZAS), “CHINA, Yunnan Prov., Longling Co., Longjiang, Xiaoheishan, riverside, 24.82888N, 98.76001E, 2020 m, 2005.V.26 N, Kavanaugh D. collector”. 1 female (IZAS), “Yunnan, Yingjiang county, Famuchang, 1770 m, 1980.IV.16, Li Hongxing leg”.

#### Diagnosis.

Medium body size, length 8.8–10.8 mm; dorsal surface black, elytra with faint cupreous hue; anterior patch zigzag, three to five intervals in width; when widest, occupying the third to seventh intervals; posterior patch separated, composed of four small spots, in intervals 2–3, 4–5, 6, and 7–8 respectively. Pronotum transverse, PW/PL 1.55–1.60; lateral margins sinuate before posterior angles; disc with very fine wrinkles. Elytra flat; apex slightly curved; outer apical angles rounded; sutural angles blunt; third interval with three setigerous pores, the middle one at approximately anterior three-fifth; eighth and ninth intervals with sparse fine setae. Median lobe of aedeagus strongly dilated, total length / greatest width approximately 3.7 (Fig. 35).

#### Comparison.

This species is most similar with *P. obscuratus* sp. n. in the genus. For comparisons between them, see Comparison section of that new species. *Pericalus obtusipennis* is also very similar to *P. distinctus* from India and Myanmar. Detailed discussions on these two species are provided below.

*Pericalus obtusipennis* might be confused with *P. ornatus* or *P. acutidens* due to their similar elytra pattern and sympatric distribution. But *P. obtusipennis* is different from the latter two species in several external characters: (1) elytral outer apical angles rounded in *P. obtusipennis*; acuminate, acute or obtuse in other two species; (2) elytral anterior patch zigzag in *P. obtusipennis*, always very narrow and strongly transverse (similar to that in *P. acutidens*); but rounded or nearly rounded in *P. ornatus*, rarely somewhat zigzag, but wider; (3) in *P. obtusipennis*, elytral posterior patches always with a separate small spot on the sixth interval, placed much beyond to that in the fourth and fifth intervals (Fig. 15); in the other two species, spot in sixth interval usually absent (Figs 9, 10); if present, adjacent to the larger spot in the fourth and fifth interval (Figs 11, 12); (4) all three species with very faint metallic hue on elytra, but cupreous in *P. obtusipennis* and cyan in the other two species. Moreover, *P. obtusipennis* has the median lobe of the aedeagus strongly dilated, very different from those of all other known species.

From the elytral pattern and outer angle, *P. obtusipennis* is also similar to *P. amplus* with which it is sympatric with in Yunnan and N. Vietnam. These two species can be distinguished by: (1) *P. obtusipennis* with three setigerous pores in elytral third interval; *P. amplus* with four pores; (2) wrinkles along inner margin of eyes a little coarser and sparser in *P. obtusipennis*, with 7–8 wrinkles on each side; a little finer and denser in *P. amplus*, with 9–10 wrinkles on each side; (3) elytra a little more convex in *P. amplus*; (4) male genitalia very different (Figs 35, 40).

#### Supplemental description.

*Male genitalia* (Fig. 35). Median lobe of aedeagus rather stout, total length / greatest width approximately 3.7 (in lateral view), strongly dilated after basal bend, and abruptly narrowed before apical lamella; ventral margin nearly straight in the middle, dorsal margin evenly curved; apical orifice large, reaching one third length of the median lobe, opened to the left; apical lamella small, digitiform, length approximately 1.5 times as basal width; endophallus with fine scales all through length, without spines. *Female genitalia*. Internal reproductive system (Fig. 50): spermatheca pedunculate, inserted on the joint of common oviduct and bursa copulatrix; spermathecal body fusiform, longer than the pedicel; spermathecal gland inserted on the joint of spermathecal pedicel, apex shortly dilated. Gonocoxite 2 of ovipositor (Fig. 49) scimitar-shaped, abruptly bent to the outer side at apical fourth; length approximately six times as basal width; outer margin with four dorsolateral ensiform setae, the basal one much finer than the other three; inner margin with one doromedial ensiform seta near apex.

#### Distribution.

Vietnam (Lao Cai), China (Yunnan) (Fig. 60). This species is sympatric with *P. acutidens* sp. n. in west Yunnan, but seems to prefer a higher elevation (1700–2000 m) and is much rarer.

#### Remarks.

The little known species *P. distinctus* Dupuis, recorded from India and Myanmar, is very similar to *P. obtusipennis*. From the very brief original description (Dupuis 1913) and comments added later (Andrewes 1937), diagnostic characters of *P. distinctus* can be summarized as follows: elytral outer apical angles rounded or obtuse; elytral anterior patch composed of a transverse band, in intervals third to sixth, slightly obliquely backwards on each side; posterior patch similar to that of *P. ornatus*; third interval with three setigerous pores; legs and antennae pale reddish brown, much lighter than *P. ornatus*. Most of these characters agree with those of *P. obtusipennis*, except for pale legs and antennae. We did not examine any confirmed material of *P. distinctus* in the present study and cannot compare it with *P. obtusipennis* further. But, with the very distinctive male genitalia, our examined specimens from Yunnan can be readily identified as *P. obtusipennis*.

### Pericalus (s. str.) obscuratus sp. n.

Taxon classificationAnimaliaColeopteraCarabidae

http://zoobank.org/4154488C-C584-4004-83FE-2534E004AD09

Figs 13 , 14 , 18 , 28 , 36 , 44 , 55 , 60

#### Type material.

**Holotype** (IZAS): male, body length = 11.1 mm, board mounted, genitalia dissected and deposited in micro vial pinned under specimen, “CHINA, Guizhou Prov., Jiangkou county, Fanjingshan Mt. S. slope, 4500 steps (Huixiangping) to 5300 steps (ropeway tower); N27.90180 E108.70372 – N27.90784 E108.70052; 1778–1973m, 2012.VIII.24, night, on tree trunk, broadleaf forest; SHI Hongliang, HUANG Xinlei, LIU Yizhou leg., Inst. of Zoo., CAS”; “HOLOTYPE ♂ Pericalus obscuratus sp. n., des. SHI & LIANG 2018” [red label]. (Fig. 18) **Paratypes** (10 ex.): **Guizhou**: 1 male, 2 females (IZAS): same data as holotype. 2 females (IZAS): same data as holotype but date 2012.VIII.25. 1 female (IZAS), “CHINA, Guizhou Prov., Jiangkou county, Fanjingshan Mt. S. slope, 5300 steps to upper ropeway station); N27.90784 E108.70052 – N27.91027 E108.69846; 1973–2078 m, 2012.VIII.25, night, on dead log, broadleaf forest; SHI Hongliang, HUANG Xinlei, LIU Yizhou leg., Inst. of Zoo., CAS”. 2 males and 1 female (CCCC), “China, Guizhou prov., Fanjingshan mt., Huixiangping, N27.9018, E108.7037, 1775 m, 2009.VI.22, Lin W.S. lgt.”; **Guangxi**: 1 female (CCCC), “CHINA, Guangxi, Jinxiu, Dayaoshan, Fenzhanshan, 1050m, 2017.IV-13, J.-T. Zhao leg. CCCC”.

#### Diagnosis.

Medium size, body length 9.9–11.5 mm; dorsal surface black, elytra with very faint cyan hue; anterior patch zigzag, three to five intervals in width, when widest, in the third to seventh intervals; posterior patch separate, composed of four small spots, in intervals 2–3, 4–5, 6, and 7; pronotum transverse, PW/PL 1.60–1.75; lateral margins barely sinuate before posterior angles; elytral outer apical angles rounded, sutural angles blunt; third interval with three setigerous pores; median lobe of aedeagus a little dilated.

#### Comparison.

This new species is very similar to *P. obtusipennis*. It can be distinguished from the latter species by: (1) all elytral yellowish patches smaller than *P. obtusipennis*, most of patches equal or shorter than the interval width in *P. obscuratus* sp. n., usually longer than the interval width in *P. obtusipennis*; (2) elytra with very faint cyan metallic hue; with faint cupreous hue in *P. obtusipennis*; (3) pronotum usually wider (PW/PL = 1.60–1.75) than in *P. obtusipennis* (PW/PL = 1.55–1.60); (4) pronotal lateral margins barely sinuate before posterior angles (versus slightly but distinctly sinuate); (5) in *P. obscuratus* sp. n., median lobe of aedeagus only a little dilated, in lateral view total length / greatest width approximately 4.8 (Fig. 36); strongly dilated in *P. obtusipennis*, total length / greatest width approximately 3.7 (Fig. 35). In spite of the above characters, these two species are almost identical, but their different male genitalia support that they are distinct species.

#### Description.

Body length 9.9–11.5 mm. *Coloration*. Dorsal surface black, elytra with very faint cyan hue, with yellowish patches; mouthparts, antennomeres 2–11 reddish brown; legs dark brown, tarsi yellowish brown; ventral side black. Elytral anterior patch zigzag, in the third to seventh intervals, pale markings in the third and seventh intervals sometimes faint; pale markings in odd intervals anteriorly placed, those in even intervals posteriorly placed; the zigzag patch narrow and a little oblique, usually subequal to single interval width, pale marking in the fifth interval a little anterior to that in the third. Elytral posterior patch separate, composed of four small spots in intervals 2–3, 4–5, 6, and 7; each spot usually shorter than single interval width; from anterior to posterior, the order of four spots: 6, 4–5, 7, 2–3. *Microsculpture* faint and isodiametric on vertex and pronotum disc, distinct and linear on elytral intervals. *Head* densely rugose; three to five shallow wrinkles on each side extending from clypeus to frons; eight to ten fine wrinkles along each side of inner margin of eye, reaching level of posterior margin of eyes; vertex and occiput almost smooth. Eyes strongly prominent; temporae gradually constricted behind eyes. *Pronotum* strongly transverse, PW/PL = 1.60–1.75, sub-equal to the width of head with eyes (PW/HW = 1.00–1.11); posterior margin close to the width of anterior margin; lateral margins rounded in the middle, very weakly sinuate before posterior angles; posterior angles nearly rectangular, not or weakly projecting laterally, with a seta very close to the posterior angles; lateral expansions wide; disc weakly convex, nearly smooth, with very faint wrinkles, with a pair of very shallow pits on each side; sub-anterior impression barely visible, median line fine, not reaching anterior nor posterior margin; basal fovea shallow, extended medially merged with the shallow sub-posterior impression, extended posteriorly forming very shallow short oblique grooves. *Elytra* ovate, weakly convex; EW/EL = 0.72–0.74; much wider than pronotum, EW/PW = 1.51–1.63; apical truncation weakly curved; outer apical angles rounded, sutural angles blunt; striae moderately incised, impunctate; third interval with three setigerous pores, the first one at approximately basal eighth, the second at approximately apical two fifth, the third one close to apex; the first one adjacent to the third stria, the other two close to the second stria; intervals slightly convex, the eighth interval tumid apically, the eighth and ninth intervals with sparse fine seta aside of umbilical series; lateral expansions narrowly extended, a little widened near basal third. *Male genitalia* (Fig. 36). Median lobe of aedeagus a little dilated, total length / greatest width approximately 4.8 (in lateral view), gradually narrowed before apical lamella; ventral margin unevenly curved near middle, dorsal margin evenly curved; apical orifice large, reaching one fourth length of the median lobe, opened left-ventrally; apical lamella small, gradually narrowed to apex, length approximately 1.5 times as basal width; endophallus with fine scales all through length, without spines. Right paramere with apex extended and enlarged, securiform. *Female genitalia*. Internal reproductive system (Fig. 55): spermatheca pedunculate, inserted on the joint of common oviduct and bursa copulatrix; spermathecal body digitiform, longer than the pedicel, distinctly bent; spermathecal gland inserted on the joint of spermathecal pedicel, apex not dilated (probably spermathecal gland apex missing), nearly same length as spermathecal body. Gonocoxite 2 of ovipositor (Fig. 44) scimitar-shaped, abruptly bent to the outer side at apical fifth; length approximately six times as basal width; outer margin with three dorsolateral ensiform setae, the basal one finer than and a little distant from the rest two; inner margin with one dorsomedial ensiform seta near apex.

#### Distribution.

Only known from two isolated localities in southern China: Fanjingshan (Guizhou) and Dayaoshan (Guangxi) (see Fig. 60).

#### Etymology.

This name *obscuratus* means indistinct, referring to the elytral pattern of this new species which is much narrower than those of all other allied species, almost indistinct.

#### Habitat.

In Fanjingshan, this species occurs in evergreen and deciduous broad-leaved mixed forests, with dominant trees of *Cyclobalanopsis* spp. and *Fagaus* spp., with an elevational range of 1775 to 2078 m. Adults were collected on or under bark of dead trees during the night.

#### Remarks.

The new species is closest to *P. obtusipennis*. Their male genitalia, although different in thickness, are of the same form: somewhat dilated, large apical orifice, and small apical lamella.

##### Examined material of *Pericalus* (*s. str.*) species not recorded from China

*Pericalus baehri* Fedorenko: 1 male (IZAS), “Gunung Leser National Park, Sumatra, local collector, 2014”.

*Pericalus cicindeloides* Macleay: 22 males and females (IZAS), “Gunung Leser National Park, Sumatra, local collector, 2014”.

*Pericalus cordicollis* Andrewes: 1 male (IZAS), “Borneo: Sabah, Ranau distr., Kinabalu Park, Liwagu Trail, N6.0239, E116.5500, 1704m; Shi H.L. & Liu Y. leg. Dead log, 2016.V.5d”.

*Pericalus funestus* Andrewes: paratype, 1 male (NNML), “Gunung Singgalang, Sumatra’s Westkust, *1800*M. *VII* 192, leg. E. Jacobson.”, “*Cotype*”, “*Pericalus funestus Cotype Andr*., H.E. Andrewes det.”, “Museum Leiden, *Pericalus funestus andr.*, Det *Andrewes*”, “type”.

*Pericalus guttatus* Chevrolat: syntype, 1 male (OUM), “TYPE *COL*: *128 Pericalus guttatus Chevr.*, HOPE DEPT. OXFORD”, “Chevrolat Carabidae. Fr.V.d.Poll. Pres. 1909, E. B. Poulton.”

*Pericalus longicollis* Chaudoir: 3 males, 1 female (IZAS), “Gunung Leser National Park, Sumatra, local collector, 2014”; 2 males (IZAS), “Borneo: Sabah, Keningau district, Jungle Girl Camp, N5.4430, E116.4512; 1182m, Shi H.L & Liu Y. leg., light trap, 2016.IV.25N”.

## Supplementary Material

XML Treatment for Pericalus (s. str.)

XML Treatment for Pericalus (s. str.) gibbosus

XML Treatment for Pericalus (s. str.) dux

XML Treatment for Pericalus (s. str.) elegans

XML Treatment for Pericalus (s. str.) amplus

XML Treatment for Pericalus (s. str.) acutidens

XML Treatment for Pericalus (s. str.) ornatus ornatus

XML Treatment for Pericalus (s. str.) ornatus formosanus

XML Treatment for Pericalus (s. str.) obtusipennis

XML Treatment for Pericalus (s. str.) obscuratus
